# Phylogeny of Myzostomida (Annelida) and their relationships with echinoderm hosts

**DOI:** 10.1186/s12862-014-0170-7

**Published:** 2014-08-28

**Authors:** Mindi M Summers, Greg W Rouse

**Affiliations:** Scripps Institution of Oceanography, UCSD, 9500 Gilman Drive, La Jolla, CA 92093 USA

**Keywords:** Co-phylogeny, Parasitism, Symbiosis, Taxonomy, Systematics, *Asteromyzostomum*, *Contramyzostoma*, *Eenymeenymyzostoma*, *Myzostoma*, *Protomyzostomum*

## Abstract

**Background:**

Myzostomids are marine annelids, nearly all of which live symbiotically on or inside echinoderms, chiefly crinoids, and to a lesser extent asteroids and ophiuroids. These symbionts possess a variety of adult body plans and lifestyles. Most described species live freely on the exterior of their hosts as adults (though starting life on the host inside cysts), while other taxa permanently reside in galls, cysts, or within the host’s mouth, digestive system, coelom, or gonads. Myzostomid lifestyles range from stealing incoming food from the host’s food grooves to consuming the host’s tissue directly. Previous molecular studies of myzostomids have had limited sampling with respect to assessing the evolutionary relationships within the group; therefore molecular data from 75 myzostomid taxa were analyzed using maximum likelihood and maximum parsimony methods. To compare relationships of myzostomids with their hosts, a phylogeny was inferred for 53 hosts and a tanglegram constructed with 88 associations.

**Results:**

Gall- and some cyst-dwellers were recovered as a clade, while cyst-to-free-living forms were found as a grade including two clades of internal host-eaters (one infecting crinoids and the other asteroids and ophiuroids), mouth/digestive system inhabitants, and other cyst-dwellers. Clades of myzostomids were recovered that associated with asteroids, ophiuroids, and stalked or feather star crinoids. Co-phylogenetic analyses rejected a null-hypothesis of random associations at the global level, but not for individual associations. Event-based analyses relied most upon host-switching and duplication events to reconcile the association history.

**Conclusion:**

Hypotheses were revised concerning the systematics and evolution of Myzostomida, as well their relationships to their hosts. We found two or three transitions between food-stealing and host-eating. Taxa that dwell within the mouth or digestive system and some cyst forms are arguably derived from cyst-to-free-living ancestors – possibly the result of a free-living form moving to the mouth and paedomorphic retention of the juvenile cyst. Phylogenetic conservatism in host use was observed among related myzostomid taxa. This finding suggests that myzostomids (which have a free-living planktonic stage) are limited to one or a few closely related hosts, despite most hosts co-occurring on the same reefs, many within physical contact of each other.

**Electronic supplementary material:**

The online version of this article (doi:10.1186/s12862-014-0170-7) contains supplementary material, which is available to authorized users.

## Background

**Figure 1:**
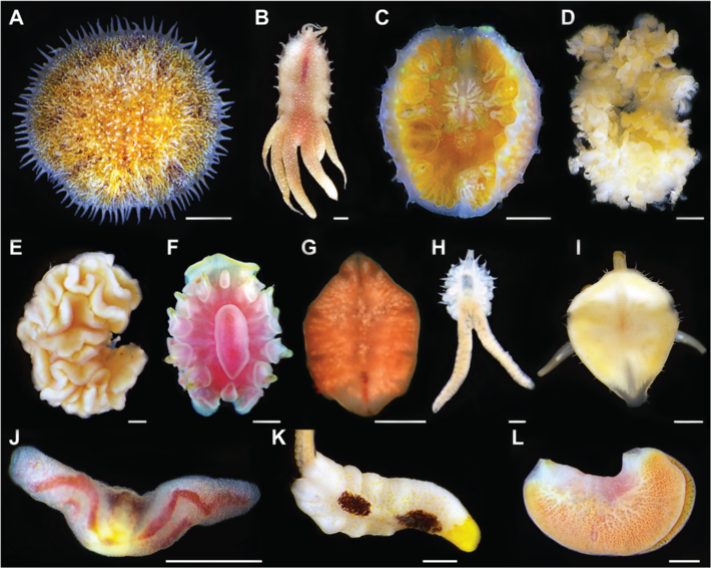
**Diversity of Myzostomida. (A)** *Myzostoma capitocutis* (Myzostomatidae), free-living. **(B)** *Myzostoma* eeckhauti *nomen nudum* (Summers & Rouse, *in press*.) (Myzostomatidae), free-living. **(C)** *Pulvinomyzostomum* inaki *nomen nudum* (Summers & Rouse, *in press*.) (Pulvinomyzostomatidae), found in or near mouth. **(D)** *Mesomyzostoma* sp. (Myzostomatidae, previously Mesomyzostomatidae), resides within the host’s coelom*.* **(E)** *Asteromyzostomum* grygieri *nomen nudum* (Summers & Rouse, *in press*.) (Asteromyzostomatidae), recovered externally with mouth pierced through body wall. **(F)** *Notopharyngoides aruensis* (Myzostomatidae), found in mouth. **(G)** *Protomyzostomum* roseus *nomen nudum* (Summers & Rouse, *in press*.) (Protomyzostomatidae)*,* found within the host’s coelom. **(H)** *Myzostoma divisor* (Myzostomatidae), free-living*.* **(I)** *Notopharyngoides platypus* (Myzostomatidae), resides permanently in cysts. **(J)** *Contramyzostoma bialatum* (Myzostomatidae, previously Endomyzostomatidae), resides permanently in cysts. **(K)** *Myzostoma longitergum* (Myzostomatidae), free-living. **(L)**. *Endomyzostoma* neridae *nomen nudum* (Summers & Rouse, *in press*.) (Endomyzostomatidae), lives within galls. A, D, I, K—Raja Ampat, Indonesia. B, J—Madang Habor, Papua New Guinea. C—Costa Rica. E, H—Antarctica. F—Lizard Island, Australia. G—Monterey, California. L—Dili, East Timor. Scale bars 1 mm (A, D-G, I-L); 0.5 mm (H); 0.2 mm (B, C).

Myzostomida Graff, 1877 is a clade of marine annelid worms with around 150 described species. Most spend their adult lives living on or inside crinoid echinoderms; about a dozen species are associated with ophiuroids and asteroids; two occur on black coral (Antipatharia) [1,2]; and one has been recorded from a sponge [3]. Myzostomids occur in shallow subtidal to abyssal depths throughout all of the world’s oceans, but are mainly found in shallow-water tropical reefs where crinoid diversity is greatest [4,5]. Myzostomids possess a variety of body plans and lifestyles in which they steal food from or directly consume the host (Figure 1). Those that live on the surface of the animal are mainly disk-shaped or elongated; they use their chaetae to hold onto the host while inserting their proboscis into the host’s food groove to steal food [4,6]. These external types may “mimic” the host by possessing similar colors and/or having appendages that resemble parts of the crinoid, for example, extensions that look like pinnules [6–8]. Other myzostomids reside in galls (hard) or cysts (soft) along the host’s food grooves, or within the host’s mouth, digestive system, coelom, or gonads [4–6,9]. Those living on the outside of the animal and within galls, cysts, the mouth, and digestive track are presumed to be stealing the host’s food, while those within the coelom and gonads are believed to be eating the host directly [4,6].

Though once controversial, molecular and morphology analyses now support myzostomids as part of the annelid radiation, but the relationship of Myzostomida to other annelid groups remains unresolved [10–16]. Three published phylogenies have treated relationships within Myzostomida. The first two are based solely on morphology [6,17], and the most recent is based on molecular data (three genes) from 37 taxa [18]. Grygier [6] and Lanterbecq et al. [18] revealed problems with the classification within the group, though no changes from the system proposed by Jägersten [17] (Table 1, left column) have yet been made. The molecular phylogeny of Lanterbecq et al. [18] recovered two major clades, one composed of gall-, cyst-, and mouth-dwelling taxa associated with crinoids, and the other including mostly free-living taxa, along with cyst, mouth, and internal forms. Based on this phylogeny, they proposed the ancestral state for Myzostomida to have been an external form found on crinoids and argued for independent emergences of gall, cyst, and internal forms [18,19].

**Table 1:** **Classifications of Myzostomida Graff, 1877**

**Previous classification**	**Revised classification**
Pharyngidea Jägersten, 1940	*Asteriomyzostomatidae Jägersten, 1940
Asteriomyzostomatidae Jägersten, 1940	*Asteriomyzostomum Jägersten, 1940 (2 species)
*Asteriomyzostomum* Jägersten, 1940	Asteromyzostomatidae Wagin, 1954
Asteromyzostomatidae Wagin, 1954	*Asteromyzostomum* Wagin, 1954 (3 species)
*Asteromyzostomum* Wagin, 1954	Eenymeenymyzostomatidae Summers & Rouse
Endomyzostomatidae Perrier, 1897	*Eenymeenymyzostoma* Summers & Rouse (1 species)
*Contramyzostoma* Eeckhaut & Jangoux, 1995	Endomyzostomatidae Perrier, 1897
*Endomyzostoma* Perrier, 1897	*Endomyzostoma* Perrier, 1897 (14 species)
*Mycomyzostoma* Eeckhaut, 1998	Myzostomatidae Beard, 1884
Mesomyzostomatidae Stummer-Traunfels, 1923	^*Contramyzostoma* Eeckhaut & Jangoux, 1995 (1 species)
*Mesomyzostoma* Remscheid, 1918	*Hypomyzostoma* Perrier, 1897 (10 species)
Protomyzostomatidae Stummer-Traunfels, 1923	*^Myzostoma* Leuckart, 1827 (115 species)
*Protomyzostomum* Fedetov, 1912	*Mesomyzostoma* Remscheid, 1918 (2 species)
Pulvinomyzostomatidae Jägersten, 1940	*^Notopharyngoides* Uchida, 1992 (3 species)
*Pulvinomyzostomum* Jägersten, 1940	Protomyzostomatidae Stummer-Traunfels, 1923
Stelechopodidae Graff, 1884	*Protomyzostomum* Fedetov, 1912 (4 species)
*Stelechopus* Graff, 1884	Pulvinomyzostomatidae Jägersten, 1940
Proboscidea Jägersten, 1940	*Pulvinomyzostomum* Jägersten, 1940 (1 species)
Myzostomatidae Beard, 1884	*Stelechopodidae Graff, 1884
*Hypomyzostoma* Perrier, 1897	*Stelechopus Graff, 1884 (1 species)
*Myzostoma* Leuckart, 1827	Family uncertain
*Notopharyngoides* Uchida, 1992	*Mycomyzostoma Eeckhaut, 1998 (1 species)

**Figure 2:**
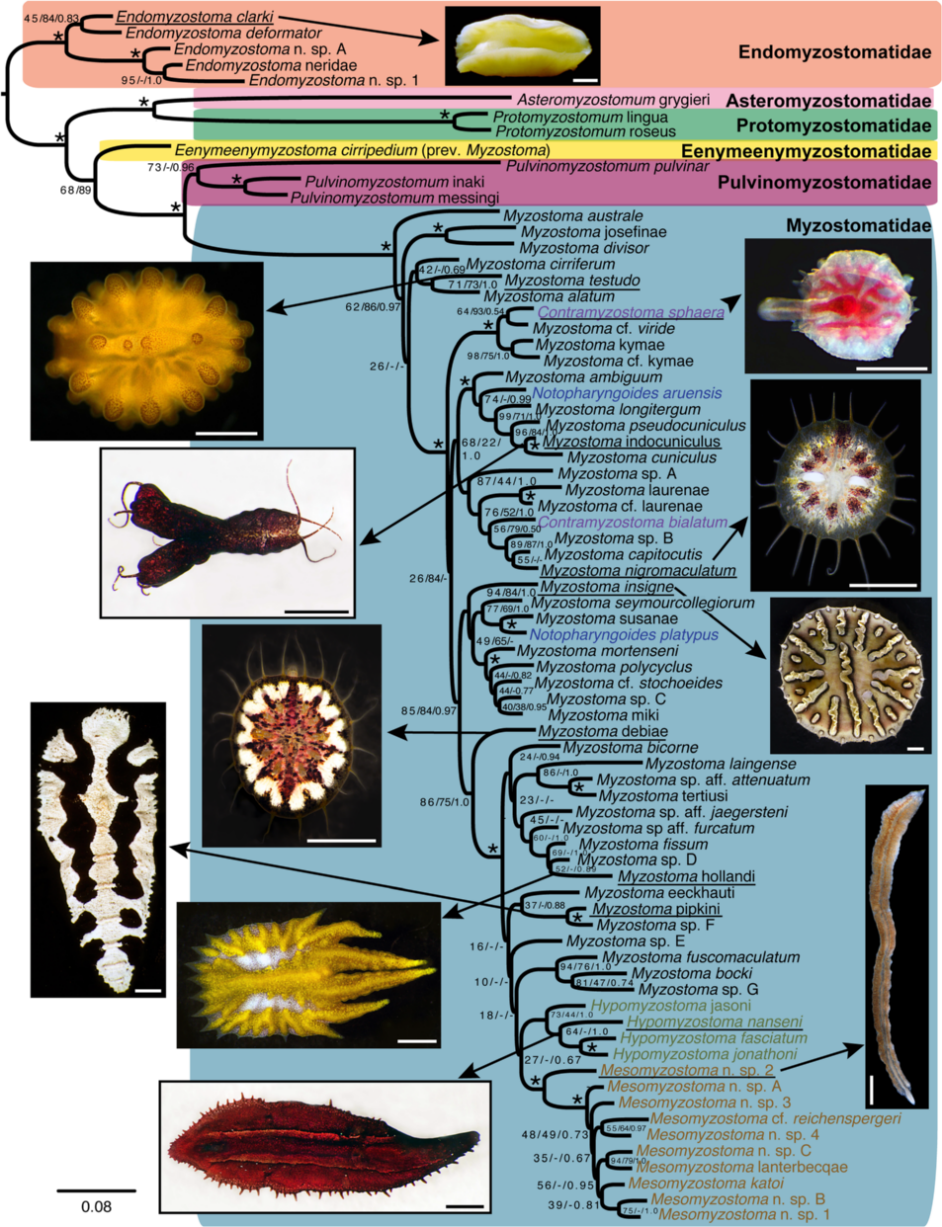
**Phylogeny of Myzostomida.** Maximum likelihood (ML) tree inferred from the concatenated four-gene dataset, all positions included. Symbols near nodes refer to bootstrap (BS) and jackknife (JK) support scores and posterior probabilities (PP), for ML, maximum parsimony (MP), and Bayesian analyses, respectively. An asterisk indicates nodes with >90% bootstrap and jackknife support and >0.95 posterior probability. Other scores are represented BS/JK/PP. A hyphen is given for nodes not recovered in MP or Bayesian analyses. Colored boxes surround clades delimited in the taxonomic revision provided herein. Photographs correspond to underlined taxa. Scale bars 1 mm. Specific names written in plain text are for species described in a separate publication – Summers & Rouse, *in press*. These names are disclaimed for nomenclatural purposes under ICZN 8.3 and are not made available through this publication.

Previous classification (Jägersten [17]; Grygier [6]) shown in left column. Classification as revised here, with the number of currently described species for each genus, shown in the right column. * indicate groups with no molecular data available. ^ denote paraphyletic/polyphyletic taxa based on the molecular phylogeny in Figure 2.

The variety of life histories combined with dependence on an echinoderm host makes Myzostomida an interesting lineage in which to explore the evolutionary effects of obligate symbiosis and relationships with their echinoderm hosts. Signs of suspected myzostomid infestations have been found on fossilized stalked crinoids from the early Jurassic [20] and possibly as far back as the Silurian [21]. A previous coevolutionary analysis of sixteen hosts and myzostomids recovered significant cophylogeny [22].

In this paper we sample a substantially broader range of myzostomid species with accompanying DNA sequences, allowing a reassessment of the phylogeny and evaluation of symbiotic lifestyles, host specificity, and host affinity. We revise the classification of Myzostomida accordingly and re-examine congruence between the phylogeny of myzostomids and their hosts.

## Results

### Phylogeny of Myzostomida

Additional file 1: Table S1 lists the nominal species and terminals used to assess myzostomid phylogeny, including the genes sequenced for this study and those obtained from GenBank. The maximum parsimony (MP) and maximum likelihood (ML) results for each gene partition are summarized in Additional file 1: Table S2. Results from individual genes were basically congruent with each other, and with the combined data analyses (MP, ML, and Bayesian), and are not shown here. The aligned sequence data from the four genes (COI, 16S, 18S, H3) yielded a concatenated ‘complete’ dataset of 3019 characters with 1187 parsimony informative sites and 252 variable, but uninformative, sites. The ‘restricted’ dataset (with 16S, 18S, and H3 Gblocked and third positions of COI excluded) had 2501 characters, with 908 parsimony informative sites and 182 variable, but uninformative, sites.

The ML and Bayesian analyses of the complete (ML = −ln 38806.84) and restricted (ML = −ln 26899.17) datasets gave the same tree topology (Figure 2). *Endomyzostoma* Perrier, 1897 (living in galls or cysts on crinoids) formed a well-supported clade sister to all other myzostomids. Within this latter clade, *Asteromyzostomum* Jägersten, 1940 (associated with a seastar) was found with high support as sister to *Protomyzostomum* Fedetov, 1912 (living in ophiuroids) and this clade was then sister to the remaining myzostomids found on crinoids. *Myzostoma cirripedium* Graff, 1885, a free-living form on a stalked crinoid (previously found within *Endomyzostoma* and referred to this genus [18]), was sister to the well-supported clade of *Pulvinomyzostomum* Jägersten, 1940 and the remaining myzostomids. The placement of *Myzostoma cirripedium* requires that it be placed in a new genus, *Eenymeenymyzostoma* n. gen. (see Taxonomy section). *Pulvinomyzostomum*, with type species *Pulvinomyzostomum pulvinar* (Graff, 1884), formed a clade (through likelihood, but not parsimony analyses) with two other undescribed myzostomid species. *Pulvinomyzostomum* inaki *nomen nudum* (Summers and Rouse *in press*) has the same lifestyle as *P. pulvinar* (in the mouth of the crinoid), while *Pulvinomyzostomum* messingi *nomen nudum* (Summers and Rouse *in press*) is a free-living form on its stalked hyocrinid host.

The MP analysis of the complete dataset yielded 100 most parsimonious trees, length 9386. The MP analysis of the restricted dataset yielded 56 most parsimonious trees with a tree length of 5451. The MP results were similar to each other and largely congruent with the ML and Bayesian analyses, but the grade of taxa shown in Figure 2 with respect to Myzostomatidae, was instead a grade with respect to *Endomyzostoma* (Additional file 1: Figure S1). Also, the three species forming the *Pulvinomyzostomum* clade in the ML and Bayesian analyses were not monophyletic in the MP results. Two basic topologies were considered for the interpretation of the transformations, the first ML- and Bayesian- based (Figure 2) and the second MP-based (Additional file 1: Figure 1).

With regard to the relationships within the well-supported Myzostomatidae, all MP, ML, and Bayesian analyses showed *Myzostoma* Leuckart, 1827 as paraphyletic with the genera *Contramyzostoma* Eeckhaut & Jangoux, 1995, *Hypomyzostoma* Perrier, 1897, *Mesomyzostoma* Remscheid, 1918 and *Notopharyngoides* Uchida, 1992 nested inside (Figure 2, Additional file 1: Figure S1). Relationships in the MP analyses varied among the species of *Myzostoma* in the various shortest trees for the complete and restricted analyses and between these analyses and the ML and Bayesian analyses. This is reflected in the poor support for many of the nodes across Myzostomatidae (Figure 2). What all analyses did show was that *Myzostoma australe* Rouse, 2003 was the sister group to all other Myzostomatidae.

The ML and Bayesian analyses presented a monophyletic *Hypomyzostoma* as a poorly supported sister-group to a well-supported *Mesomyzostoma* clade (Figure 2), as did some MP trees, though with poor support. *Hypomyzostoma* has the type species *H. folium* (Graff, 1884), which was not available for this study. However, based on morphology and following Lanterbecq et al. [18], we apply the name *Hypomyzostoma* to the clade comprising four nominal species (*H.* jonathoni *nomen nudum* (Summers & Rouse, *in press.*), *H.* jasoni *nomen nudum* (Summers & Rouse, *in press*), *H. nanseni* (Graff, 1887), and *H. fasciatum* (Remscheid, 1918)).

All results recovered *Contramyzostoma* as polyphyletic, with *C. sphaera* Eeckhaut, Grygier, & Deheyn, 1998 as the sistergroup to *Myzostoma* cf. *viride,* and *C. bialatum* Eeckhaut & Jangoux, 1995, the type species of the genus, as sister to another clade of *Myzostoma* species. *Notopharyngoides* was also consistently polyphyletic, with *N. aruensis* (Remscheid, 1918) and *N. platypus* (Graff, 1887) well separated and sister groups to different clades of *Myzostoma*.

With regards to family-ranked taxa, the concatenated ML and Bayesian analyses (see Figure 2) returned four of the current families as clades: Asteromyzostomatidae (monotypic), Protomyzostomatidae, Pulvinomyzostomatidae, and Mesomyzostomatidae (Table 1-left column), though the latter taxon rendered Myzostomatidae paraphyletic. The MP analyses did not recover what is delineated as Pulvinomyzostomatidae in Figure 2 as a clade (see Additional file 1: Figure S1). The concatenated ML, Bayesian, and MP analyses recovered Endomyzostomatidae as polyphyletic (two genera sequenced). *Endomyzostoma* formed a clade that was sister to all other myzostomid terminals (Figure 2) and is here retained as Endomyzostomatidae, while the two terminals of *Contramyzostoma* were nested within clades composed primarily of *Myzostoma* (Figure 2, Additional file 1: Figure S1). Myzostomatidae was recovered as paraphyletic (all three genera sequenced), owing to the placement of *Contramyzostoma* (Endomyzostomatidae) and *Mesomyzostoma* (Mesomyzostomatidae) among the various Myzostomatidae terminals, and these taxa are now referred to this family. Figure 2 and Table 1 (right column) indicate the revised family taxon memberships and names.

### Lifestyle transformations

**Figure 3:**
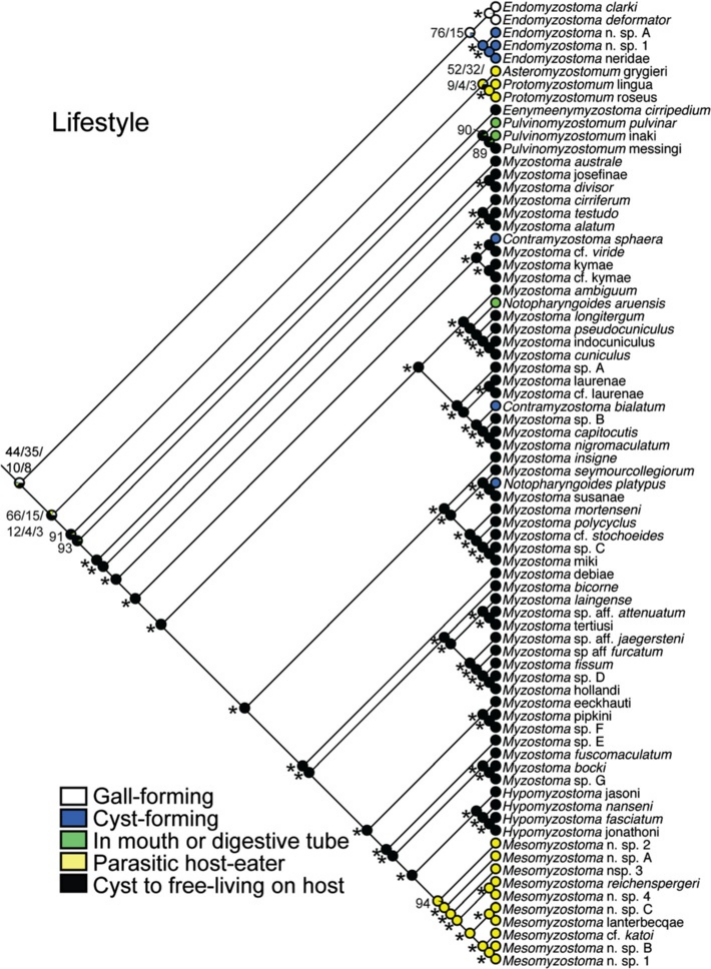
**Maximum likelihood transformations for myzostomid lifestyle.** Number and symbols near nodes refer to proportional likelihood estimations. An asterisk indicates nodes with an estimated proportional likelihood of >95%. Other scores are provided in order of most likely states and separated by a forward slash when applicable. See Figure 2 regarding non-italicized names.

Most myzostomids live externally on their host following a period of development in a cyst (i.e., cyst-to-free-living). This condition evidently arose once in both the ML/Bayesian and MP topologies, and is found in *Eenymeenymyzostoma* n. gen. and Myzostomatidae (Figure 3). Gall-forming myzostomids were recovered as a single clade within *Endomyzostoma.* Adult cyst-dwelling myzostomids were recovered in both *Endomyzostoma* and Myzostomatidae. In *Endomyzostoma*, these cyst-forming taxa were a clade. Three other adult cyst-dwellers (*Contramyzostoma sphaera*, *Contramyzostoma bialatum,* and *Notopharyngoides platypus*) were distributed across Myzostomatidae, each sharing a most recent common ancestor with a form that transitioned from a cyst to an external adult life. In all of these cases, this ancestor was estimated to have been cyst to free-living (prop. likelihood 1.00). Two taxa found in the mouth or digestive tube were in *Pulvinomyzostomum* and one in Myzostomatidae (*Notopharyngoides aruensis*), each arguably arising from a cyst to free-living ancestor (Figure 3). Host-eating forms were within *Asteromyzostomum* (found on/in asteroids), *Protomyzostomum* (living in ophiuroids), and *Mesomyzostoma* (living within crinoids). For both ML/Bayesian and MP topologies, internal host-eating was most likely the plesiomorphic condition for *Mesomyzostoma* (prop. likelihood 0.94) and possibly *Asteromyzostomum* + *Protomyzostomum* clades (prop. likelihood 0.58 and 0.52 respectively).

### Patterns of host use and specificity

**Figure 4:**
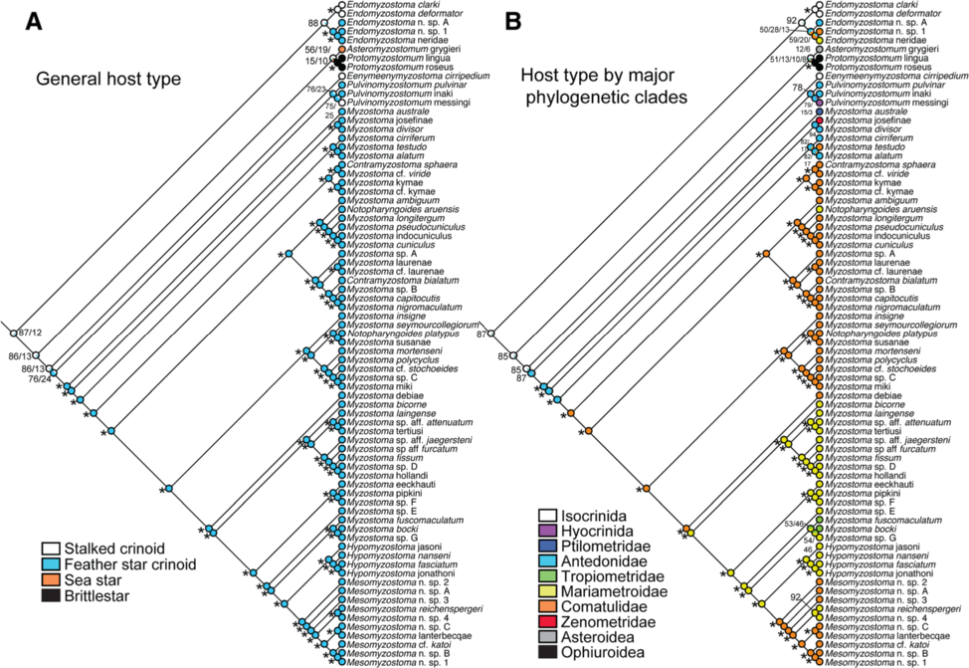
**Maximum likelihood transformations for general host type (A) and host type by major clade (B).** Symbols near nodes refer to proportional likelihood estimations. An asterisk indicates nodes with an estimated proportional likelihood of >95%. Symbols, scores, and non-italicized names as in Figure 3.

Figure 4A shows transformations for general host type. Association with asteroids and ophiuroids is restricted to *Asteromyzostomum* and *Protomyzostomum,* respectively. *Eenymeenymyzostoma* n. gen. resides exclusively on stalked crinoids, and the sequenced Myzostomatidae only inhabit feather stars. *Endomyzostoma* and *Pulvinomyzostomum* infect both stalked and feather star crinoids. The ML/Bayesian topology suggests a stalked crinoid was the ancestral host for Myzostomida (prop. likelihood 0.87), with one transition to asteroids (*Asteromyzostomum*), one to ophiuroids (*Protomyzostomum*), and three transitions to feather stars (in *Endomyzostoma* and the clade of Pulvinomyzostomatidae + Myzostomatidae, with a reversal back to a stalked crinoid for *Pulvinomyzostomum* messingi *nomen nudum*). Transformations on the MP topology suggest a feather star crinoid as the ancestral host (prop. likelihood 0.87), with one transition to asteroids, one to ophiuroids, and two or three transitions to stalked crinoids (for *Pulvinomyzostomum* messingi *nomen nudum*, *Eenymeenyzostoma cirripedium,* and in *Endomyzostoma,* or *Pulvinomyzostomum* messingi *nomen nudum* and the ancestor of *E. cirripedium* + *Endomyzostoma*, with a reversal to stalked crinoids in *Endomyzostoma*). Six most parsimonious reconstructions (MPRs) were found for host type on both ML/Bayesian and MP topologies, the variation due to unresolved nodes for *Asteromyzostomum* and *Protomyzostomum*.

Figure 4B shows the maximum likelihood transformations for major host clades, which mostly correspond to family-level classification of crinoids and also include Asteroidea de Blainville, 1830 and Ophiuroidea Gray, 1840. Isocrinid stalked crinoids are hosts to gall-*Endomyzostoma* and cyst-to-free-living *Eenymeenymyzostoma* n. gen*.,* while *Pulvinomyzostomum* messingi *nomen nudum* is found on a hyocrinid stalked crinoid*.* Among feather stars, species of Antedonidae Norman, 1865 host two *Pulvinomyzostomum* taxa and two Myzostomatidae taxa. *Myzostoma australe* is found on a Ptilometridae AH Clark, 1914 and *M. divisor* Grygier, 1989 and *Myzostoma* josefinae *nomen nudum* (Summers & Rouse, *in press*.) on Zenometridae AH Clark, 1909*.* Comatulidae Fleming, 1828, Mariametroidea AH Clark, 1909, and Tropiometridae AH Clark, 1908 all host taxa within Myzostomatidae. The large number of MPRs for both ML/Bayesian and MP topologies (132 and 66 respectively) suggest multiple scenarios regarding switches among major host clades, especially at the family-level for myzostomids. Within Myzostomatidae, both topologies suggest two transitions to association with Comatulidae, three to four switches to Mariametroidea*,* and one or two inhabitations of Tropiometridae.

Additional file 1: Figure S2 presents the distribution of host specificity on the ML/Bayesian topology. Occurrence on only one host is most common, and is present in all families except Eenymeenymyzostomatidae n. fam. The 12 MPRs suggest that infestation of more than one host arose multiple times independently.

### Comparison of myzostomid and host phylogenies

**Figure 5:**
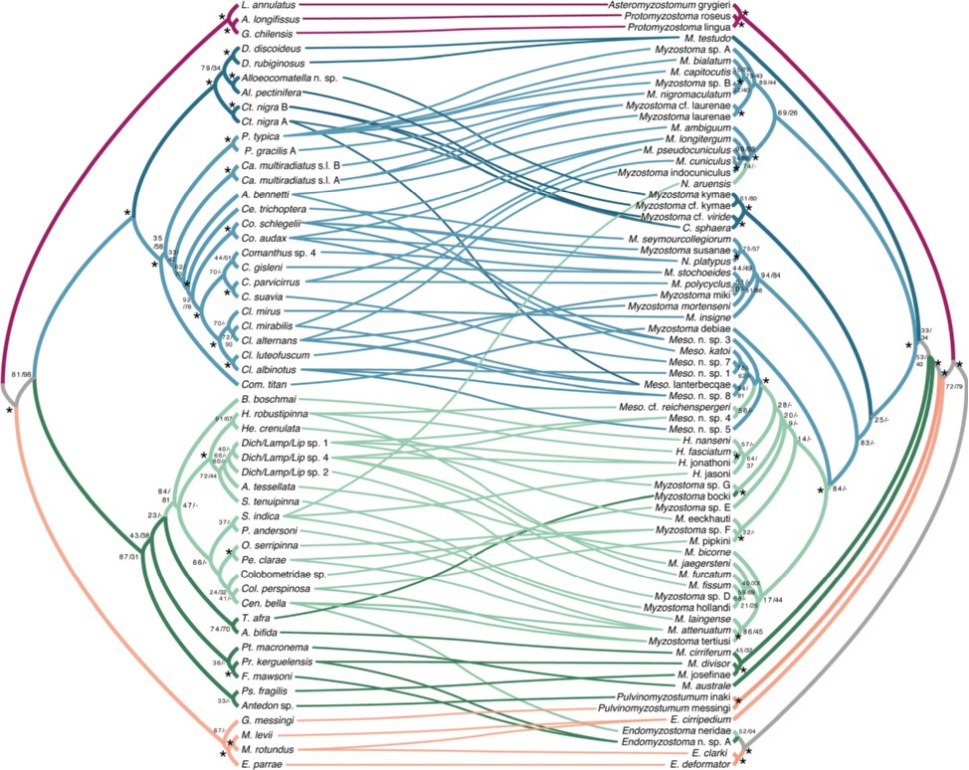
**Associations of myzostomids with their echinoderm hosts.** Tanglegram of 88 associations among host (left side) and myzostomid (right side) phylogenies. Symbols near nodes refer to bootstrap (BS) and jackknife (JK) support scores, for ML and maximum parsimony (MP) analyses, respectively. Symbols, scores, and non-italicized names as in Figure 3.

Figure 5 shows associations among myzostomids and their hosts (88 links in total). The host was known for 69 of 75 myzostomid terminals (Additional file 1: Table S1). Additional file 1: Table S3 lists the nominal species and 53 terminals used to estimate the host phylogeny. The MP and ML results for the concatenated datasets and each gene partition are provided in Additional file 1: Table S2. The overall phylogeny of the 69-myzostomid phylogeny was congruent with that recovered for all included terminals. For hosts, there were four major well-supported clades: stalked crinoids (Isocrinida and Hyocrinida), asteroids and ophiuroids, one family of feather stars (Comatulidae), and all other feather stars (Figure 5).

Most clades of myzostomids were associated with specific clades of hosts (branches shown in the same color – Figure 5). Exceptions include *Pulvinomyzostomum* (the two included terminals associate with a stalked and feather star crinoid, respectively), *Notopharyngoides aruensis, Mesomyzostoma* (taxa are found within two clades of feather stars), and *Endomyzostoma* (specimens infest stalked crinoids and two clades of feather stars). *Endomyzostoma* species are known from all the crinoid groups referenced here (as in Figure 4B) and along with *Pulvinomyzostomum* and *Mesomyzostoma,* are likely considerably undersampled.

Parafit analysis of myzostomids and their hosts rejected the null hypothesis of random association (ParaFitGlobal = 2439.93156; P = 0.00050). Three of the 88 individual links were significant (for terminals of *Asteromyzostomum* and *Protomyzostomum*) (prob1 and prob2 < 0.016). JANE analyses under default settings led to 4297 reconstructions (cost = 139) for the ML/Bayesian-rooted topology (e.g., Figure 5). Duplication with host switch was most used (40–41), followed by 29–30 losses, 19 failures to diverge, 18 co-speciation events, and 9–10 duplications. The mean costs were estimated as 377 (STD 30) for random associations and 331 (STD 25) for random parasite topology.

## Discussion

### Taxonomic implications

This molecular study incorporated 75 myzostomid terminals representing six of the eight subfamilies and nine of the 12 genera. Previously, Lanterbecq et al. [18] offered the only hypothesis for myzostomid evolution based on molecular data. They incorporated three genes (one nuclear, two mitochondrial) for 37 species from nine myzostomid genera, and recovered taxa associated with ophiuroids (members of Protomyzostomatidae) within Mesomyzostomatidae [18], a family otherwise containing species living in the coelom and gonads of crinoids. They also found Mesomyzostomatidae and a terminal attributed to *Asteromyzostomum* (an associate of an asteroid) nested among free-living terminals of Myzostomatidae [18].

Our results recovered four of the families as monophyletic: Asteromyzostomatidae, Protomyzostomatidae, Pulvinomyzostomatidae, and Mesomyzostomatidae (Table 1-left column). *Mesomyzostoma* was recovered within Myzostomatidae as in the phylogeny of Lanterbecq et al. [18], but *Asteromyzostomum* and *Protomyzostomum* were instead recovered as a well-supported clade sister to *Pulvinomyzostomum* and Myzostomatidae. We sequenced recently collected *Asteromyzostomum* specimens from the same locality and host species and *Protomyzostomum* specimens from similar hosts to those used in Lanterbecq et al. [18]. Since the previously sequenced material was old and the amplified sequences were short and very similar to other reads amplified in the same study, we suspect that these previous sequences are inaccurate. Amended identifications for these, and other specimens sequenced in Lanterbecq et al. [18] are provided in the caption of Additional file 1: Table S1.

Molecular results from ML, MP, and Bayesian analyses recovered both myzostomid families that have more than one genus, Endomyzostomatidae and Myzostomatidae, as paraphyletic. We restrict Endomyzostomatidae to include only *Endomyzostoma* and transfer *Contramyzostoma* to Myzostomatidae (Table 1, right column). Eeckhaut and Jangoux [23] assigned *Contramyzostoma* to Endomyzostomatidae based on its cyst-dwelling lifestyle (see [6]). *Contramyzostoma* possess a salivary gland arrangement resembling that of *Myzostoma,* and Grygier [6] suggested their morphological affinity to Myzostomatidae. Also, cyst forms of *Endomyzostoma* may be gonochoric and have been found with dwarf males, while those of Myzostomatidae are generally simultaneous hermaphrodites and have long protrusible penes to inseminate mates in nearby cysts (see [6,19]). To make Myzostomatidae monophyletic, *Mesomyzostoma* (Mesomyzostomatidae) is also moved to this family (Table 1, right column), resulting in Mesomyzostomatidae becoming a junior synonym of Myzostomatidae. To accommodate *Myzostoma cirripedium* Graff, 1885*,* a species recovered well outside all other members of *Myzostoma*, *Eenymeenymyzostoma* n. gen. and Eenymeenymyzostomatidae n. fam. are erected here (see Taxonomy section).

This study recovered four genera including more than one terminal as monophyletic (*Endomyzostoma, Mesomyzostoma, Pulvinomyzostomum,* and *Protomyzostomum*), one genus as paraphyletic (*Myzostoma*) (polyphyletic before erection of *Eenymeenymyzostoma* n. gen. for *Myzostoma cirripedium*), and two genera as polyphyletic (*Contramyzostoma* and *Notopharyngoides*). Although *Myzostoma* is clearly paraphyletic, this genus will not be revised here. The type species for *Myzostoma* is *M. cirriferum* Leuckart, 1836, which was recovered as part of a relatively basal grade of terminals in Myzostomatidae (Figure 2). There was little support in this region of the topology and there was conflict among the various ML, Bayesian, and MP analyses. Revising the membership of *Myzostoma* to make it monophyletic based on Figure 2 would restrict the genus to just a few species, and so require name changes for all the remaining species currently in the genus. We refrain from doing so until a more robust phylogeny is obtained.

The polyphyly found here for the cysticolous *Contramyzostoma* (two species) and *Notopharyngoides* (three species) is not unexpected*.* The morphology of *Contramyzostoma sphaera* differs considerably from the type species, *C. bialatum,* and Eeckhaut et al. [24] only tentatively assigned it to this genus. As we are not fully revising the taxonomy within Myzostomatidae, we simply remove *C. sphaera* from the same genus as *C. bialatum* and refer to it as *Myzostoma sphaera*. Sequenced taxa of *Notopharyngoides* differ in the location of the proboscis as well as where they live on the host. The proboscis is ventral in *N. platypus*, which resides in cysts on the oral surface, and dorsal in *N. aruensis*, which occupies the mouth. We did not have sequences of the type species, *Notopharyngoides ijimai* (Hara & Okada, 1921), which has a dorsal proboscis, and we thus leave *Notopharyngoides* as polyphyletic pending further study.

### Evolution of lifestyles

Taxa that steal the host’s food (e.g., cyst-to-free-living, cyst, gall, and mouth/digestive forms) were recovered as a grade (Figure 3). Mouth and digestive-system dwellers (some *Pulvinomyzostomum,* some *Notopharyngoides*) and permanent cyst forms within Myzostomatidae (*Contramyzostoma,* some *Notopharyngoides)* were arguably derived from cyst-to-free-living ancestors. These permanent cyst-forms may have arisen via paedomorphosis, since, as far as is known for myzostomid development, free-living forms initially develop in a cyst [6]. Observations of mouth-dwelling taxa are consistent with a free-living myzostomid relocating to a stationary position in the mouth. Females of *Pulvinomyzostomum pulvinar* occupy the stomach, esophagus, and mouth, while smaller males are found either on the body of the female or free-living on the host [17].

Forms in which the adults consume the host directly have arguably arisen either twice independently (Figure 3) – in the ancestor of *Asteromyzostomum* and *Protomyzostomum* (which reside in asteroids and ophiuroids) and in the ancestor of *Mesomyzostoma* (which live within crinoids) – or three times (separately in *Asteromyzostomum* and *Protomyzostomum*). Finding and studying internal myzostomids requires dissection of the host, and their diversity is likely considerably undersampled. Lanterbecq et al. [18] considered internal and external host-eaters, gall and cyst food-stealers, and some stationary free-living forms as parasites; as a result, they suggested multiple independent switches to ‘parasitism’. Although all myzostomid forms could be called parasites, differentiating host-eaters and food-stealers as done here reduces the emergence of host consumption (which in some cases can lead to castration (e.g., [25,26]) to two or possibly three times during the evolution of myzostomids.

The morphology and lifestyle of the ancestral myzostomid form is not clear, especially when the closest annelid relative remains elusive. Likelihood estimates for the ancestral node for the ML/Bayesian-rooted topology were split between gall and cyst-to-free-living forms, with cyst and internal forms also likely. Based on this transformation, a free-living lifestyle (following development in a cyst) arose once on a deep-sea stalked crinoid (Figures 3). When considering the MP-rooted topology, a free-living form is estimated for the ancestral node, as suggested in previous studies [18,19].

### Patterns of host associations

High host specificity and phylogenetic conservatism in host use was observed (Additional file 1: Figure S2; Figure 4), the latter likely contributing to the non-random pattern of associations among hosts and myzostomids. Myzostomids have a planktonic larval stage [27,28], and their crinoid hosts may occur in multi-species assemblages in close physical proximity [29]. These two factors make this result surprising, suggesting that myzostomids are restricted to certain echinoderms and switch mainly to evolutionarily related hosts. Such a pattern of phylogenetic conservatism in host use is widespread among a variety of parasites and hosts, including gall-inducing insects (see [30]), brood parasites (e.g., [31,32]), fish parasites [33], and many others (see [34] for a general review). Specificity in this system may be promoted through a number of mechanisms. Myzostomids may only be capable of infesting certain echinoderm groups, but little is currently known about recruitment and subsequent development of myzostomid larvae. In experimental studies, Eeckhaut and Jangoux [27] observed the host crinoid (*Antedon bifida*) expelling *Myzostoma cirriferum* larvae from its food grooves using its podia and also regurgitating ingested larvae. They did find that *M. cirriferum* larvae could recruit onto the crinoid at a specific developmental stage. A comparative study of recruitment and reproductive success across a range of myzostomids and their hosts could be a first step in understanding whether or not myzostomids are equally capable of infesting a range of crinoids, or rather if they have specialized tactics for better success on certain species.

Examination of the co-phylogenetic structure of Myzostomida and their hosts does not support strict topological congruence. Although the global null hypothesis of independent association using ParaFit was rejected, only three links out of the 88 associations were significantly concordant. These links were for myzostomids associated with asteroids and ophiuroids. Rather than a signal of ‘co-speciation,’ this congruence is likely an artifact of the small number of known asteroids and ophiuroids that are infected by myzostomids. In event-based analyses, host switches and duplications were mostly used to reconcile the myzostomid and host trees, numbering 40–41 compared to 18 for co-speciations. Acknowledging that event reconstructions are best used for exploratory purposes rather than explanatory ones (and that we did not perform an exhaustive exploration of possible cost schemes or topologies), the high number of events other than ‘co-speciation’ and the discordance found for the majority of links using distance methods, leads us to infer that this system is not a model of strict phylogenetic ‘co-speciation.’ Global concordance could have resulted from a number of mechanisms, including, but not limited to, host tracking, vicariance, and co-speciation (see [35,36] for reviews). Inferring the mechanisms underlying this pattern requires experimental follow-up.

## Taxonomic changes

### Eenymeenymyzostomatidae n. fam. Summers & Rouse

*Included genera. Eenymeenymyzostoma* n. gen. Summers & Rouse.

### *Eenymeenymyzostoma* n. gen. Summers & Rouse

*Type species. Myzostoma cirripedium* Graff, 1885 – Sagami Bay, Japan, 218 m.

*Etymology.* This name was chosen for its assonance (suggested by Charles Messing).

*Remarks.* To accommodate *Myzostoma cirripedium* Graff, 1885*,* a species recovered well outside all other members of *Myzostoma*, *Eenymeenymyzostoma* n. gen. and Eenymeenymyzostomatidae n. fam. are erected here. *Eenymeenymyzostoma* n. gen*.* is currently monotypic. Sequences for this species were referred to as *Endomyzostoma* n. sp. 2 in Lanterbecq et al. [18]. *Myzostoma metacrini* McClendon, 1906 is a junior synonym of *E. cirripedium*. Other potential members of *Eenymeenymyzostoma* n. gen. include ten other free-living myzostomid species described from stalked crinoids (excluding *Pulvinomyzostomum* messingi *nomen nudum* (Summers & Rouse, *in press*)); molecular data are not currently available for these taxa*.*

### Endomyzostomatidae Perrier, 1897

*Included genera. Endomyzostoma* Perrier, 1897

*Remarks.* Endomyzostomatidae as previously delineated (including *Contramyzostoma, Endomyzostoma,* and *Mycomyzostoma*) was recovered as polyphyletic in the molecular analyses (e.g., Figure 2). To make Endomyzostomatidae monophyletic, it is restricted here to include only *Endomyzostoma. Contramyzostoma* is transferred to Myzostomatidae and the placement of *Mycomyzostoma* is uncertain until specimens are sequenced.

### Myzostomatidae Beard, 1884

*Included genera. Contramyzostoma* Eeckhaut & Jangoux, 1995; *Hypomyzostoma* Perrier, 1897; *Myzostoma* Leuckart, 1827; *Mesomyzostoma* Remscheid, 1918; *Notopharyngoides* Uchida, 1992.

*Remarks.* To make Myzostomatidae monophyletic, *Contramyzostoma* (previously Endomyzostomatidae) and *Mesomyzostoma* (previously Mesomyzostomatidae) are transferred to this family. This results in Mesomyzostomatidae becoming a junior synonym of Myzostomatidae.

### *Contramyzostoma* Eeckhaut & Jangoux, 1995

*Type species. Contramyzostoma bialatum* Eeckhaut & Jangoux, 1995

*Remarks.* The two previous members of *Contramyzostoma, C. bialatum* and *C. sphaera* were not recovered within a single clade. To eliminate this polyphyly, we remove *C. sphaera* from the same genus as *C. bialatum* and refer to it as *Myzostoma sphaera*. As *Myzostoma* remains non-monophyletic (see Figure 2), *Contramyzostoma* will likely incorporate more members once this genus is resolved (e.g., *Myzostoma nigromaculatum, Myzostoma capitocutis, Myzostoma* sp. A, *Myzostoma* sp. B, and *Myzostoma* laurenae *nomen nudum* (Summers & Rouse, *in press*).

### Note concerning specific names

Specific names written in plain text within this work are disclaimed for nomenclatural purposes under ICZN 8.3 and are not made available through this publication.

## Conclusions

Increased sampling and re-examination of previous work revised previous hypotheses concerning the systematics and evolution of Myzostomida, as well their relationship to their hosts. Contrary to previous reports of multiple switches among lifestyles [18], we found two or three transitions between food-stealing and host-eating. Taxa that dwell within the mouth or digestive system and some permanent cyst forms are arguably derived from cyst-to-free-living ancestors – possibly the result of a free-living form moving to the mouth and paedomorphic retention of the juvenile cyst. We recovered clades of myzostomids delimited more or less by their host-association. Phylogenetic conservatism in host use was observed for related myzostomid taxa, suggesting that myzostomes can only inhabit certain, and in most cases related, hosts.

## Methods

### Taxon sampling

Myzostomids and their hosts were collected chiefly using scuba. Animals collected at depths greater than 20 m were recovered using trawls or remotely operated vehicles (ROVs). Myzostomids were fixed in formalin, while hosts were preserved in ethanol or dried. Tissue subsamples of both myzostomids and hosts were placed in 95% ethanol or 20% DMSO buffer [37]. Specimens were deposited at or obtained from the American Museum of Natural History (AMNH), Australian Museum, Sydney, Australia (AMS); Muséum National d’Histoire Naturelle, Paris, France (MNHN); South Australian Museum, Adelaide, Australia (SAM); Benthic Invertebrate Collection, Scripps Institution of Oceanography, La Jolla, CA (SIO-BIC). Additional file 1: Table S1 and S3 lists locality details, voucher information, and host identifications. Live photographs were taken via Leica MZ8 or MZ9.5 stereomicroscopes with Canon Rebel T2i, T3i or T4i cameras and Speedlight flashes.

### DNA extraction, amplification, and sequencing

Genomic DNA was extracted from tissue subsamples using the Qiagen DNAeasy Tissue Kit following manufacturer protocols. For myzostomids, we sequenced four genes previously used for myzostomids (e.g., [18,25,38]). Two nuclear markers—18S rRNA (complete, approximately 1780 bp) and histone H3 (partial, approximately 350 bp), and two mitochondrial markers—16S rRNA (partial, approximately 550 bp) and Cytochrome oxidase subunit 1 (COI) (partial, approximately 700 bp). For hosts, four markers were sequenced: one nuclear—28S rRNA (incomplete, approximately 800 bp), and three mitochondrial—16S rRNA (partial, approximately 550 bp), Cytochrome oxidase subunit 1 (COI) (partial, approximately 1100 bp), and Cytochrome B (CytB) (partial, approximately 800 bp) following procedures of Rouse et al. [39]. PCR mixtures contained 12.5 μL ProMega GoTaq Green DNA polymerase (3 mM MgCl_2_, 400 μM each dNTP, 1U Taq) and between 50–100 ng DNA.

For myzostomids, the complete 18S rRNA nuclear gene (18S) for myzostomids was amplified in three overlapping fragments with the primer pairs: (1) 1 F (5’-TAC CTG GTT GAT CCT GCC AGT AG-3’) and 5R (5’-CTT GCC AAA TGC TTT CGC-3’) (950 bp); (2) 3 F (5’-GTT CGA TTC CGG AGA GGG A-3’) and bi (5’-GAG TCT CGT TCG TTA TCG GA-3’) (900 bp); (3) a.20 (5’-ATG GTT GCA AAG CTG AAA C-3’) and 9R (5’-GAT CCT TCC GCA GGT TCA CCT AC-3’) (850 bp) [40]. The reaction profiles were (1 and 3) 95°C for 180 s, 40 cycles of 95°C for 30s, 49°C for 30s, and 72°C for 90s, and finally 72°C for 480 s; (2) 95°C for 180 s, 40 cycles of 95°C for 30s, 52°C for 30s, and 72°C for 90s, and finally 72°C for 480 s. H3 was amplified using the primer pair H3F (5’- ATG GCT CGT ACC AAG CAG ACV GC -3’) and H3R (5’- ATA TCC TTR GGC ATR ATR GTG AC -3’) [41] with a reaction profile of 95°C for 180 s, 38 cycles of 95°C for 30s, 53°C for 45 s, and 72°C for 45 s, and finally 72°C for 300 s. COI was amplified using the primer pair LCO1490 (5’-GGT CAA CAA ATC ATA AAG ATA TTG G-3’) and HCO2198 (5’-ACT TCA GGG TGA CCA AAA AAT CA-3’) (700 bp) [42]. The reaction profile was 95°C for 180 s, 5 cycles of 95°C for 40s, 45°C for 40s, and 72°C for 50s, 40 cycles of 95°C for 40s, 51°C for 40s, and 72°C for 50s, and finally 72°C for 300 s. 16S rRNA was amplified using the primer pair ar (5’-CGC CTG TTT ATC AAA AAC AT-3’) and br (5’-CCG GTC TGA ACT CAG ATC ACG T-3’) [43] using a reaction profile of 95°C for 180 s, 35 cycles of 95°C for 40s, 50°C for 40s, and 68°C for 50s, and finally 68°C for 300 s. PCR products were purified with ExoSAP-IT (GE Healthcare, Uppsala, Sweden), and sequenced by Retrogen Inc. using Applied Biosystems (ABI) 3730xl DNA analyzers. Overlapping sequence fragments were assembled using Geneious version 5.5.7 created by Biomatters (available from http://www.geneious.com).

### Alignment and assessment of saturation

Sequences of each gene were aligned using the L-INS-i method of MAFFT 7.110 (Multiple Alignment using Fast Fourier Transform) [44]. The 16S, 18S, and H3 partitions for myzostomids and 16S, 18S, and 28S partitions for crinoids were assessed for ambiguous areas of alignment using the GBlocks server, and questionable areas of alignment were removed using the least stringent settings [45,46]. Substitution saturation was tested for the protein-coding genes (COI and CytB) using DAMBE 5.0.80 [47–49]. When third codon positions were assessed independently assuming a symmetric topology, the third positions of both COI datasets and CytB were found to be substantially saturated or non-informative for phylogenetics (i.e., Iss > Iss.c or Iss < Iss.c p > 0.002). Following this assessment, datasets were concatenated for myzostomids and crinoids to include (where applicable) COI and CytB with third codon removed, and, 16S, 18S, 28S, and H3 with ambiguous areas of the alignment excluded, as determined by GBlocks. To serve as a comparison, a full dataset was concatenated which includes all data. Alignments for each individual gene partition and the two final concatenated datasets are available at TreeBase (www.treebase.org).

### Rooting

The previous major study by Lanterbecq et al. [18] included a range of terminals in their alignments to serve as a root for Myzostomida. These included members of Rotifera, Platyhelminthes and Annelida. Recent studies (e.g., [10,11]) have confirmed previous views that Myzostomida are in fact members of Annelida [14]. We used here a selection of terminals from Phyllodocida, which morphological data strongly suggests contains the closest relatives of Myzostomida [14], as well as a representative of Amphinomida (Additional file 1: Table S3; Additional file 1: Figures S1 and S3), as the terminals to root the analyses, with the amphinomid *Chloeia flava* as the actual root.

With respect to the phylogenetic analyses of the echinoderm hosts, we used an asteroid and an ophiuroid in addition to those that were hosts for myzostomids, as these had a full complement of genes available (Additional file 1: Table S3). These were then pruned out of the trees for the co-phylogenetic analysis

### Phylogenetic analyses

Molecular data from a total of 75 myzostomid terminals were included in the phylogenetic analyses (Additional file 1: Table S1). Host identities for 69 of these terminals were known, and these terminals and their hosts were used in tree-estimations for the co-phylogenetic analyses.

Maximum parsimony (MP) analyses were conducted using PAUP*4.0b10 [50] with the heuristic search option for 1000 replicates using random stepwise addition of the terminals with the tree bisection reconnection permutation algorithm (TBR). Clade support was determined using 1000 jackknife replicates with 10 random additions per iteration [51].

jModeltest2 was used to determine the most suitable model of molecular evolution for each gene partition under the Akaike Information Criterion (AIC) [52,53]. This resulted in the choice of GTR + I + G for all partitions. Maximum likelihood (ML) analyses were carried out using RAxML (7.4.2) [54] with the RAxML GUI v. 0.93 [55] under the GTR + G and GTR + I + G models of substitution. The data were partitioned by gene and, for protein coding genes, by codon position. This resulted in six partitions for myzostomids and nine partitions for hosts. A thorough bootstrap analysis was carried out with 1000 pseudoreplicates using the same model for the full analysis and for individual gene analyses. Bayesian inference was conducted for each dataset using Mr.Bayes 3.2.2 [56], with two independent Metropolis coupled analyses (runs), each using four Markov chains of 50 million generations. Model choice for each partition was based on the jModeltest2 results. Run convergence was analyzed using Tracer v1.5 [57]; the first 10 million generations (10,000) trees were discarded as burn-in. The two runs for each dataset of 40,000 trees each (80,000 total) were used to generate a majority rule tree with posterior probabilities.

### Transformations

We mapped seven traits relating to life history, geography, and host specificity onto the topology of the maximum likelihood tree (Figure 2), which was mainly congruent with the Bayesian topologies. Character transformations using an Mk1 likelihood model [58] and most parsimonious reconstructions were carried out using Mesquite 2.74 [59]. Unordered, multi-state characters were used for all transformations.

*Lifestyle.* Five myzostomid lifestyles can be readily distinguished. Most described myzostomids initially develop in a cyst and are free-living on the host as adults (Figure 1A, B, H, K). Only 28 described species are not free-living as adults – 17 live in permanent galls or cysts on crinoids (Figure 1I, J, L), three reside in the mouth or digestive caeca of crinoids (Figure 1C, F, D), four penetrate the integument or digestive caeca of asteroids via their pharynx (Figure 1E), and four are found within the gonads or bursae of brittlestars (Figure 1G). Among these lifestyles, myzostomids presumably steal the host’s food or eat the host directly. Although all forms could be called parasites, we consider separately those stealing food from those eating the host directly; the latter we call ‘parasitic host-eaters.’ Lanterbecq et al. [18] interpreted some free-living taxa as commensals and an assortment of taxa as parasites (e.g., internal and external host-eaters, galls and cysts food-stealers, and some stationary free-living forms). Terminals were coded:

1. lifestyle, location on host: (0) adult in galls (hard); (1) adult in cysts (soft); (2) adult in mouth or digestive tube; (3) adult a parasitic host-eater; (4) juvenile in cyst before living externally on host.
2. food, source: (0) host’s food; (1) host itself.

*Host use.* Myzostomids are described from three echinoderm classes – asteroids, ophiuroids, and crinoids. Among crinoids, myzostomids are recorded to infect seven major clades (roughly corresponding to taxonomic families), of which four are feather stars (all represented here) and three are stalked crinoids (Bathycrinidae not represented here). Terminals were coded:

1. host, general type: (0) stalked crinoid; (1) feather star crinoid; (2) asteroid; (3) ophiuroid.
2. host, major clade: (0) Isocrinidae; (1) Hyocrinidae; (2) Ptilometridae; (3) Antedonidae; (4) Tropiometridae; (5) Mariametroidea; (6) Comatulidae; (7) Zenometridae; (7) Asteroidea; (8) Ophiuroidea.
3. specificity, number of hosts: (0) one; (1) two; (2) three.

### Comparison of myzostomid and host phylogenies

A tanglegram of associations among terminals of the myzostomid and host phylogenies was assembled using TreeMap3.0b [60] (Figure 5). Ninety associations were mapped for 69 myzostomid terminals and 53 host terminals. To evaluate possible congruence between the myzostomid and host phylogenies, we used global and event-based methods that could account for multiple associations among hosts and myzostomids. We first ran ParaFit analyses [61] on the phylogenies obtained from maximum likelihood analyses through the program CopyCat [62]. ParaFit does not require fully resolved topologies, and patristic distances were estimated from the branch lengths of the likelihood trees. 9999 replicates were conducted for statistical testing of congruence for the entire structure and individual associations. The null hypothesis tested in this method is random association of host and myzostomids. For an event-based reconciliation analysis, we used JANE [63]. In light of computational limits when working with such a large number of host and myzostomid terminals, we ran analyses only under the default cost settings. We used a generation and population size of 100 in solving the given trees, and a generation and population size of 50 in statistical estimates with random tip mapping and randomizing the parasite tree. In recognition that current methods available to explore tanglegrams were constructed for simpler host-parasite systems, we sought to use co-phylogenetic methods to investigate our data and recognize that the low support in some areas of the trees and undersampling of hosts and myzostomids has unexplored effects on the results.

### Endnote

Several taxonomic names (specific epithets) are used here that are not valid for nomenclatural purposes according to ICZN article 8.3 and are not made available through this publication. These will be formally introduced elsewhere (Summers and Rouse *in press*). These are indicated in the text by: 1. not being in italics, and, 2. being followed by the term ‘*nomen nudem*’.

## Additional file

Additional file 1: Figure S1. Maximum parsimony phylogeny of Myzostomida. **Figure S2.** Maximum likelihood transformations for number of hosts. **Figure S3.** Maximum likelihood phylogeny of Myzostomida. **Table S1.** Myzostome specimens. **Table S2.** Summary of maximum parsimony and maximum likelihood analyses. **Table S3.** GenBank accession numbers for hosts and non-myzosomid annelid outgroups used in molecular analyses.
