# Erratum: Phylogeny of Myzostomida (Annelida) and their relationships with echinoderm hosts.

**DOI:** 10.1186/s12862-015-0305-5

**Published:** 2015-03-25

**Authors:** Mindi M Summers, Greg W Rouse

**Affiliations:** Scripps Institution of Oceanography, UCSD, 9500 Gilman Drive, La Jolla, 92093 CA USA

## Taxonomic notes

Dr. Mark Grygier has informed us that the spelling of the names for four families is not correct in our paper [1]. He notes that, according to the International Code of Zoological Nomenclature [2], for genera ending in -stoma (a Greek noun), −stomatidae is correct (Art. 29.3.1), but family names for genera ending in "stomum" (a Greek noun transliterated into Latin with a change in ending), −stomidae is correct (Art. 29.3.2). Therefore the names we referred to as Asteriomyzostomatidae, Asteromyzostomatidae, Protomyzostomatidae, and Pulvinomyzostomatidae are correctly spelled as Asteriomyzostomidae, Asteromyzostomidae, Protomyzostomidae, and Pulvinomyzostomidae.

In our paper it was also drawn to our attention by Dr. Grygier that, in lacking diagnoses, we had not fulfilled the requirements of the International Code of Zoological Nomenclature [2] for erecting a new genus and family. We correct this below.

## Eenymeenymyzostomatidae n. fam. Summers & Rouse

*Type genus. Eenymeenymyzostoma* n. gen. Summers & Rouse.

*Diagnosis.* As for generic diagnosis.

*ZooBank LSID.* urn:lsid:zoobank.org:act:928336E8-6798-4E69-9C6E-148AABC08E30

## *Eenymeenymyzostoma* n. gen. Summers & Rouse

*Type species. Myzostoma cirripedium* Graff, 1885 – Sagami Bay, Japan, 218 m.

*Diagnosis.* Body ellipsoidal. Body margin smooth, with 20 cirri. Mouth and cloaca on ventral surface, anus terminal. Five pairs of parapodia with elongate parapodial cirri. Paired penes in line with third pair of parapodia. Lateral organs, alternating with parapodia.

*ZooBank LSID.* urn:lsid:zoobank.org:act:8C18B8A1-E18B-48C8-A6D1-659729EB0E46

*Etymology.* This name was chosen for its assonance (suggested by Charles Messing).

*Remarks.* The new genus and family are erected to accommodate *Myzostoma cirripedium* Graff, 1885*,* a taxon that was recovered as sister to the well supported (unnamed) clade comprised of Pulvinomyzostomidae and Myzostomatidae (see Fig 2 in the original paper). There are currently no clear morphological apomorphies for *Eenymeenymyzostoma cirripedium,* new combination, though its ellipsoidal body shape is unusual among myzostomids. Also the parapodial cirri were noted by Graff [3] as not being previously seen in other *Myzostoma* species. However, these cirri are now known for other myzostomids (e.g., *Myzostoma polycyclus* Atkins, 1927; *Myzostoma seymourcollegiorum* Rouse & Grygier, 2005) but they are not as elongate as seen in *E. cirripedium,* new comb. *Eenymeenymyzostoma* n. gen*.* is currently monotypic. Sequences for this species were referred to as *Endomyzostoma* n. sp. 2 in Lanterbecq et al. (2006). *Myzostoma metacrini* McClendon, 1906 is a junior synonym of *E. cirripedium*. Other potential members of *Eenymeenymyzostoma* n. gen. include ten other free-living myzostomid species described from stalked crinoids, excluding *Pulvinomyzostomum messingi* Summers & Rouse, 2014 [4]; molecular data are not currently available for these taxa*.*
