# Supplementary material for: Phylogeny of Myzostomida (Annelida) and their relationships with echinoderm hosts
Source: BMC Evol Biol. 2014 Aug 28;14:170. doi: 10.1186/s12862-014-0170-7 (PMC4160548; doi:10.1186/s12862-014-0170-7)
Supplement: Additional file 1: Figure S1. — Maximum parsimony phylogeny of Myzostomida. Figure S2. Maximum likelihood transformations for number of hosts. Figure S3. Maximum likelihood phylogeny of Myzostomida. Table S1. Myzostome specimens. Table S2. Summary of maximum parsimony and maximum likelihood analyses. Table S3. GenBank accession numbers for hosts and non-myzosomid annelid outgroups used in molecular analyses. [file 12862_2014_170_MOESM1_ESM.pdf]

Figure S1. Maximum parsimony phylogeny of Myzostomida. Maximum parsimony (MP) consensus tree of 12 most parsimonious trees (length = 10589) inferred from the concatenated four-gene dataset; all positions included with non-myzostomid annelid outgroup. Numbers and symbols near nodes refer to jackknife (JK) support scores. An asterisk indicates nodes with >90% support. Unlabeled nodes are those not recovered in jackknife analyses. Specific names written in plain text are for species described in a separate publication – Summers & Rouse, *in review*. These names are disclaimed for nomenclatural purposes under ICZN 8.3 and are not made available through this publication.

Figure S2. Maximum likelihood transformations for number of hosts. Numbers and symbols near nodes refer to proportional likelihood estimations. An asterisk indicates nodes with an estimated proportional likelihood of >95%. Other scores provided in order of most likely states and separated by a forward slash when applicable. See Figure S1 regarding non-italicized names.

Figure S3. Maximum likelihood phylogeny of Myzostomida. Maximum likelihood (ML) tree (-ln = 47532) inferred from the concatenated four-gene dataset; all positions included with non-myzostomid annelid outgroup. Numbers and symbols near nodes refer to bootstrap (BS) support scores. An asterisk indicates nodes with >90% support. See Figure S1 regarding non-italicized names.

Figure S1. Maximum parsimony phylogeny of Myzostomida.

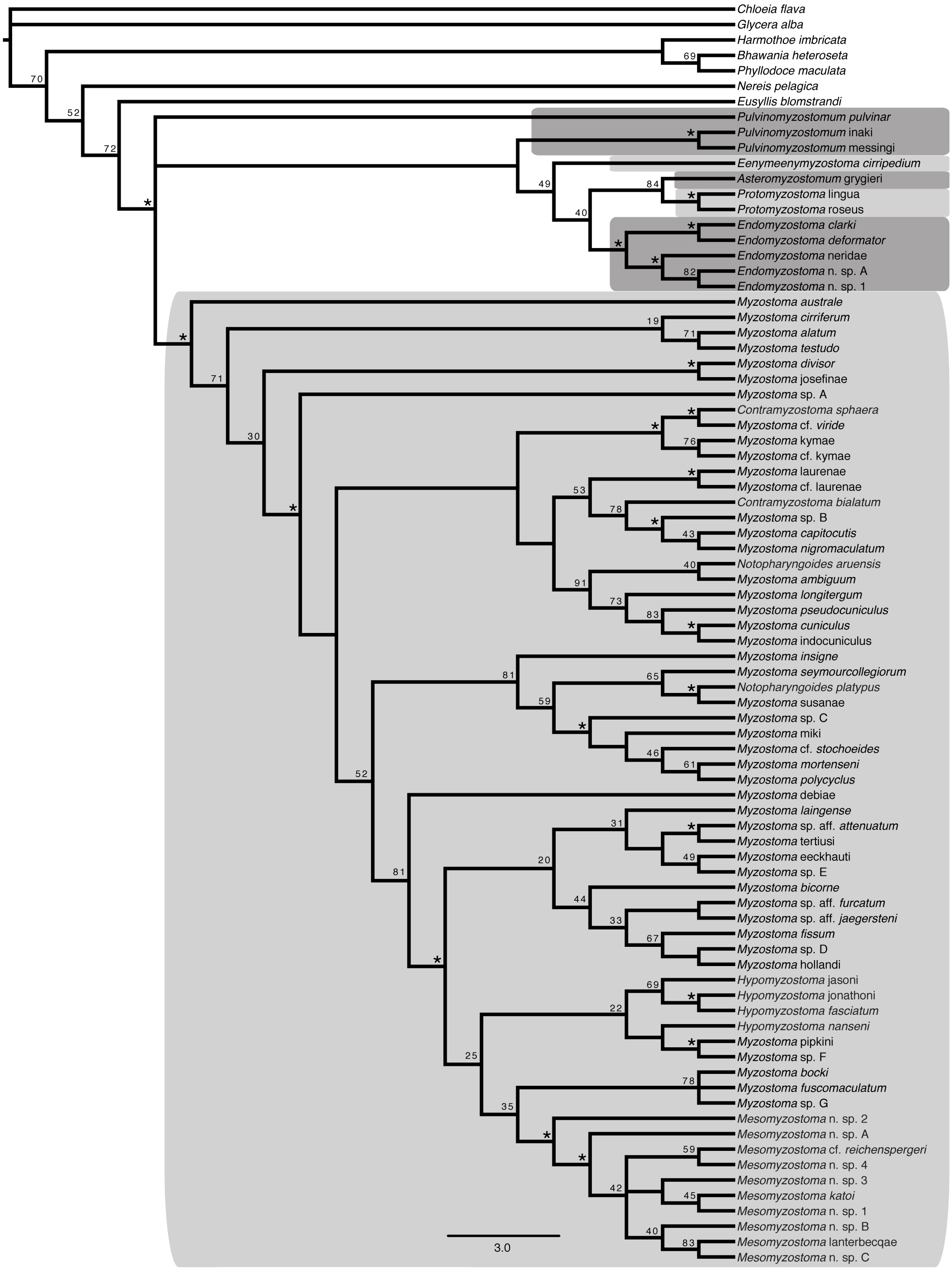

Figure S2. Maximum likelihood transformations for number of hosts.

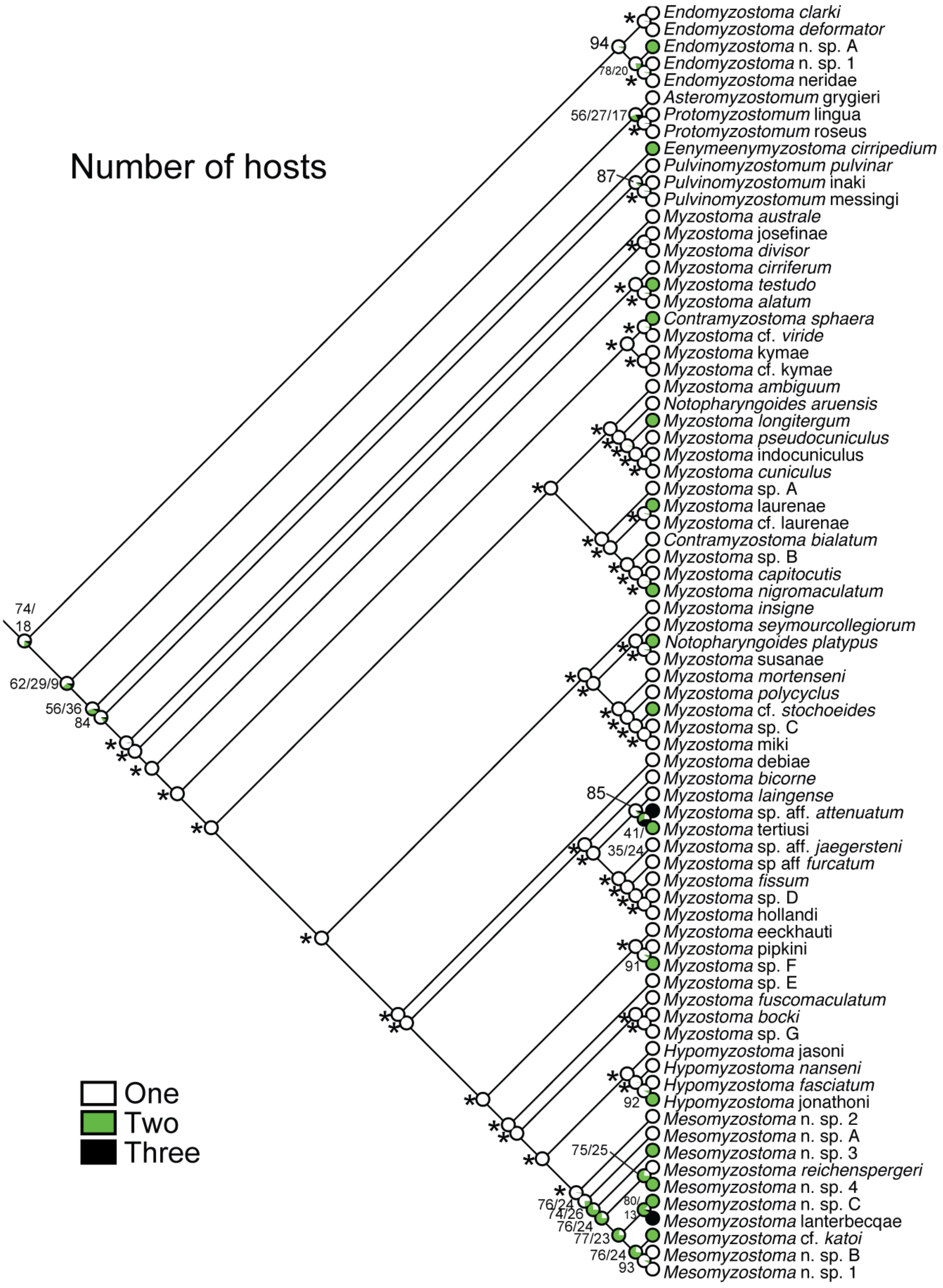

Figure S3. Maximum likelihood phylogeny of Myzostomida.

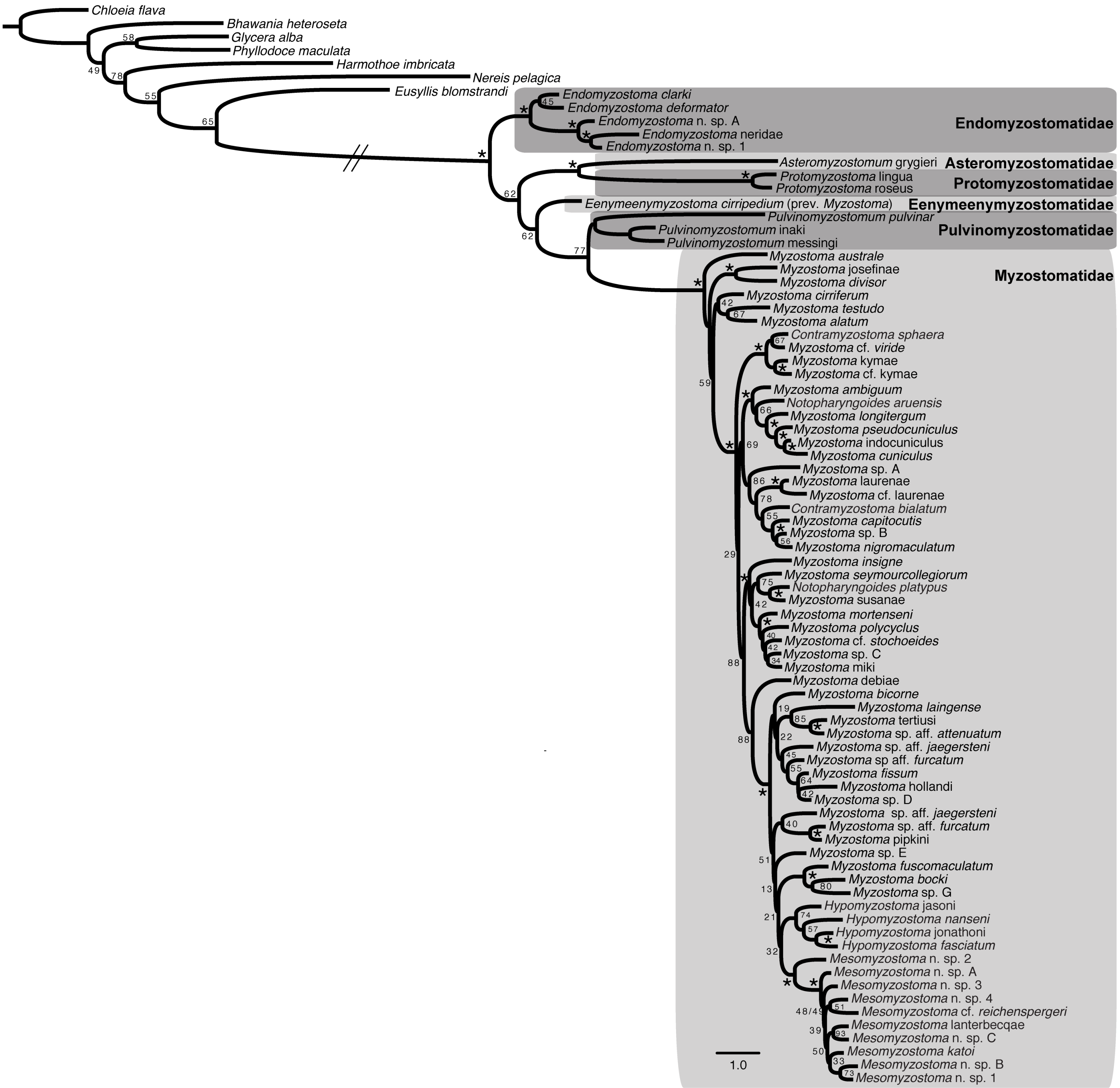

Table S1. Myzostome specimens. Collection localities, voucher information, and GenBank accession numbers for specimens used in molecular analyses. Host information only for specimens collected in this study. New sequences are in bold. Asterisks for DNA subsample only. Specific names written in plain text are for species described in a separate publication – (Summers & Rouse, *in review*). These names are disclaimed for nomenclatural purposes under ICZN 8.3 and are not made available through this publication. Mesomyzostoma sp. 1-4 are being described in Eeckhaut et al. *in prep*.

Myzostome Taxa	Voucher Accession	Host(s)	Lifestyle	Location	COI	16S	18S	H3
**Asteriomyzostomatidae** Jägersten, 1940								
Asteromyzostomum grygieri	SIO-BIC A3801	*Labidiaster annulatus*	Internal	Elephant Island, Antarctica	**KM014170**	-	**KM014252**	**KM014295**
**Eenymeenymyzostomatidae** Summers & Rouse								
Eenymeenymyzostoma cirripedium	SAM E3404	*Metacrinus rotundus* (also *Metacrinus levii*)	Free-living	Suruga Bay, Japan	DQ238191	DQ238162	DQ238127	-
**Endomyzostomatidae** Perrier, 1897								
Endomyzostoma clarki	SAM E3400	*Metacrinus* cf. *rotundus*	In galls	Japan Sea	DQ238188	DQ238159	AF260588	-
Endomyzostoma deformator	SAM E3401	*Endoxocrinus parrae*	In galls	Philippines	DQ238190	DQ238161	DQ238126	-
Endomyzostoma neridae	AM W.43447	*Cenometra bella*	In cysts	Dili, Banda Sea, East Timor	**KM014169**	**KM014214**	**KM014251**	**KM014294**
Endomyzostoma n. sp. 1	SAM E3402	Comatulidae sp.	In cysts	Toliara, Mozambique Channel, Madagascar	DQ238193	DQ238164	DQ238129	-
Endomyzostoma n. sp. A	SIO-BIC A3242	*Florometra mawsoni* (also possibly *Promachocrinus kerguelensis*)	In cysts	South Orkneys, Antarctica	**KM014168**	**KM014213**	**KM014250**	**KM014293**
**Myzostomatidae** Beard, 1884								
Contramyzostoma bialatum	SIO-BIC A3685	*Phanogenia typica*	Free-living	Madang, Bismarck Sea, Papua New Guinea	**-**	**KM014219**	**KM014257**	**KM014299**
Contramyzostoma sphaera	?/ SIO-BIC A3654	*Comatella nigra* type A; (also *Comatella nigra* type B)	In cysts	Bismarck Sea, Papua New Guinea	DQ238187	**KM014220**	AF260586	**KM014300**
Hypomyzostoma fasciatum	SAM E3396	*Himerometra robustipinna*	Free-living	Lizard Island Reef, Coral Sea, Australia	DQ238195	DQ238166	DQ238131	-

Hypomyzostoma jasoni	SAM E3398	*Stephanometra indica*	Free-living	Lizard Island Reef, Coral Sea, Australia	DQ238134	DQ238169	DQ238198	-
Hypomyzostoma jonathoni	SAM E3397	*Dichrometra/Lamprometra/Liparometra* sp. 4; (also *Dichrometra/Lamprometra/Liparometra* sp. 1)	Free-living	Lizard Island Reef, Coral Sea, Australia	DQ238197	DQ238168	DQ238133	-
Hypomyzostoma nanseni	SIO-BIC A3759	*Basilometra boschmai*	Free-living	Raja Ampat, Indonesia	**KM014183**	-	**KM014267**	-
Mesomyzostoma cf. *katoi*	SIO-BIC A3689	*Anneissia bennetti;* (also *Anneissia japonica*)	Internal	Madang, Bismarck Sea, Papua New Guinea	**KM014175**	**KM014221**	**KM014258**	**KM014301**
Mesomyzostoma lanterbecqae	SIO-BIC A3651	*Clarkcomanthus alternans;* (also *Clarkcomanthus mirabilis; Comatella nigra* type A)	Internal	Madang, Bismarck Sea, Papua New Guinea	**KM014177**	**KM014223**	**KM014260**	-
Mesomyzostoma cf. *reichenspergeri*	SAM E3410/SIO-BIC A3680	*Himerometra robustipinna*	Internal	Lizard Island Reef, Coral Sea, Australia	DQ238182	DQ238152	DQ238116	**KM014302**
Mesomyzostoma n. sp. 1	SAM E3412	*Clarkcomanthus albinotus*	Internal	Lizard Island Reef, Coral Sea, Australia	-	DQ238157	DQ238122	-
Mesomyzostoma n. sp. 2	SAM E3406	*Anneissia japonica*	Internal	Japan Sea, Japan	DQ238186	DQ238156	DQ238120	-
Mesomyzostoma n. sp. 3	SAM E3409	*Comaster schlegelii* (also *Comaster audax*)	Internal	Lizard Island Reef, Coral Sea, Australia	DQ238183	DQ238153	DQ238117	
Mesomyzostoma n. sp. 4	SAM E3408	*Dichrometra/Lamprometra/Liparometra* sp. 1*;* (also *Dichrometra/Lamprometra/Liparometra* sp. 4)	Internal	Lizard Island Reef, Coral Sea, Australia	DQ238184	DQ238154	DQ238118	-
Mesomyzostoma n. sp. A	-	*Clarkcomanthus albinotus*	Internal	Madang, Bismarck Sea, Papua New Guinea	**KM014176**	**KM014222**	**KM014259**	**KM014303**
Mesomyzostoma n. sp. B	-	*Comanthus* cf. *suavia*	Internal	Raja Ampat, Indonesia	**KM014178**	-	**KM014261**	-
Mesomyzostoma n. sp. C	SIO-BIC A3762	*Clarkcomanthus alternans;* (also *Clarkcomanthus albinotus*)	Internal	Raja Ampat, Indonesia	**KM014179**	**KM014224**	**KM014262**	**KM014304**
Myzostoma alatum	?	*Leptometra phalangium*	Free-living	Banyuls-sur-Mer, Mediterranean Sea, France	DQ238200	DQ238171	DQ238135	-

Myzostoma ambiguum	AM W.43456 /?	*Anneissia bennetti*	Free-living	Bismarck Sea, Papua New Guinea (18S); Dili, Banda Sea, East Timor	**KM014184**	**KM014228**	DQ238142	-
Myzostoma attenuatum	SIO-BIC A3715	*Cenometra bella;* (also *Petasometra* cf. *clarae; Pontiometra andersoni*)	Free-living	Madang, Bismarck Sea, Papua New Guinea	**KM014182**	**KM014227**	**KM014265**	**KM014307**
Myzostoma australe	SIO-BIC A3813	*Ptilometra macronema*	Free-living	St. Francis Isle, Nuyts Archipelago, Southern Australia	**KM014185**	**KM014229**	**KM014268**	**KM014309**
Myzostoma bicorne	SIO-BIC A3812	*Amphimetra tessellata*	Free-living	Lizard Island Reef, Coral Sea, Australia	**KM014186**	**KM014230**	**KM014269**	**KM014310**
Myzostoma bocki	SIO-BIC A3820	*Tropiometra afra*	Free-living	Kermadec Islands, Pacific Ocean, New Zealand	-	-	**KM014266**	**KM014308**
Myzostoma capitocutis	?/ SIO-BIC A3696	*Phanogenia typica*	Free-living	Bismarck Sea, Papua New Guinea	DQ238209	DQ238177	DQ238144	**KM014311**
Myzostoma cirriferum	?	*Antedon bifida*	Free-living	Morgat, Atlantic Ocean, France	DQ238199	DQ238170	AF260585	-
Myzostoma cuniculus	SAM E3393/AM W.43458	*Clarkcomanthus mirabilis*	Free-living	Lizard Island Reef (Coral Sea, Australia); Timor	DQ238203	DQ238174	DQ238138	**KM014312**
Myzostoma debiae	SIO-BIC A3794	*Comactinia titan*	Free-living	Raja Ampat, Indonesia	**KM014210**	-	**KM014291**	-
Myzostoma divisor	SIO-BIC A3216	*Promachocrinus kerguelensis*	Free-living	Shag Rocks, Antarctica	**KM014188**	**KM014232**	**KM014271**	**KM014313**
Myzostoma eeckhauti	SIO-BIC A3668	*Dichrometra/Lamprometra/Liparometra* sp. 1	Free-living	Madang, Bismarck Sea, Papua New Guinea	**KM014192**	**KM014236**	**KM014275**	**XXXX**
Myzostoma fissum	?	*Dichrometra flagellata (likely Dichrometra/Lamprometra/Liparometra* sp. 4)	Free-living	Hansa Bay, Bismarck Sea, Papua New Guinea	DQ238208	DQ238176	AF260584	-
Myzostoma sp. aff. *furcatum*	SAM E3394/SIO-BIC A3805	*Himerometra robustipinna*	Free-living	Lizard Island Reef, Coral Sea, Australia	DQ238211	DQ238178	DQ238145	**KM014315**
Myzostoma fuscomaculatum	?	*Tropiometra carinata*	Free-living	False Bay, South Africa	-	FJ346828	FJ346827	-
Myzostoma hollandi	SIO-BIC A3791	*Stephanometra tenuipinna*	Free-living	Raja Ampat, Indonesia	**KM014208**	-	**KM014289**	-

Myzostoma indocuniculus	SIO-BIC A3763	*Clarkcomanthus alternans*	Free-living	Raja Ampat, Indonesia	**KM014209**	-	**KM014290**	**KM014334**
Myzostoma insigne	SIO-BIC A3802	*Clarkcomanthus mirus*	Free-living	Lizard Island Reef, Coral Sea, Australia	**KM014191**	**KM014235**	**KM014274**	**KM014317**
Myzostoma sp. aff. *jaegersteni*	SIO-BIC A3806	*Dichrometra/Lamprometra/Liparometra* sp. 4	Free-living	Lizard Island Reef, Coral Sea, Australia	**KM014202**	**KM014244**	-	**KM014329**
Myzostoma josefinae	SIO-BIC A3798	*Psathyrometra fragilis*	Free-living	Monterey Canyon, California, USA	**KM014189**	**KM014233**	**KM014272**	**KM014314**
Myzostoma kymae	SIO-BIC A3681	*Alloeocomatella* n. sp.	Free-living	Madang, Bismarck Sea, Papua New Guinea	**KM014197**	-	**KM014280**	-
Myzostoma cf. kymae	-	*Alloeocomatella pectinifera*	Free-living	Madang, Bismarck Sea, Papua New Guinea	**KM014204**	**KM014245**	**KM014286**	-
Myzostoma laingense	?/ SIO-BIC A3816	*Stephanometra indica*	Free-living	Hansa Bay, Bismarck Sea, Papua New Guinea (COI, 18S); Lizard Island Reef, Coral Sea, Australia	DQ238205	-	DQ238141	**KM014318**
Myzostoma laurenae	SIO-BIC A3809	*Capillaster multiradiatus* s.l. A	Free-living	Lizard Island Reef, Coral Sea, Australia	**KM014198**	-	**KM014281**	**KM014326**
Myzostoma cf. laurenae	SIO-BIC A3657	*Capillaster multiradiatus* s.l. A*;* (also *Capillaster multiradiatus* s.l. B)	Free-living	Madang, Bismarck Sea, Papua New Guinea	**KM014203**	-	**KM014285**	**KM014330**
Myzostoma longitergum	SIO-BIC A3819	*Comaster schlegelii;* (also *Comaster audax*)	Free-living	Lizard Island Reef, Coral Sea, Australia	**KM014193**	-	**KM014276**	**KM014319**
Myzostoma miki	SIO-BIC A3834	*Clarkcomanthus luteofuscum*	Free-living	Great Barrier Reef, Coral Sea, Australia	**KM014201**	**KM014243**	**KM014284**	**KM014328**
Myzostoma mortenseni	?/SIO-BIC A3784	*Clarkcomanthus alternans*	Free-living	Hansa Bay, Bismarck Sea, Papua New Guinea (COI, 18S); Raja Ampat, Indonesia	DQ238206	**KM014238**	DQ238142	**KM014321**
Myzostoma nigromaculatum	SIO-BIC A3824	*Phanogenia gracilis* type A*;* (also *Phanogenia typica*)	Free-living	Madang, Bismarck Sea, Papua New Guinea	**KM014195**	**KM014239**	**KM014278**	**KM014322**
Myzostoma pipkini	SAM E3399/SIO-BIC A3818	*Colobometra perspinosa*	Free-living	Lizard Island Reef, Coral Sea, Australia	DQ238196	DQ238167	DQ238132	**KM014306**

Myzostoma polycyclus	?/ SIO-BIC A3832	*Comanthus parvicirrus*	Free-living	Hansa Bay, Bismarck Sea, Papua New Guinea (COI, 16S, 18S); Lizard Island Reef, Coral Sea, Australia	DQ238202	DQ238173	DQ238137	**KM014323**
Myzostoma pseudocuniculus	?	*Comanthus parvicirrus*	Free-living	Toliara, Mozambique Channel, Madagascar	DQ238204	DQ238175	DQ238139	-
Myzostoma seymourcollegiorum	?/SIO-BIC A3815	*Cenolia trichoptera*	Free-living	Bicheno, Tasmania, Australia	EF506562	EF506562	EF506559	**KM014324**
Myzostoma cf. *stochoeides*	SIO-BIC A3724	*Comanthus gisleni; Comanthus* sp. 4	Free-living	Madang, Bismarck Sea, Papua New Guinea	**KM014196**	**KM014240**	**KM014279**	**KM014325**
Myzostoma susanae	SAM E3879	*Comaster schlegelii*	Free-living	Lizard Island Reef, Coral Sea, Australia	**KM014200**	**KM014242**	**KM014283**	**KM014327**
Myzostoma tertiusi	SIO-BIC A3703	*Cenometra bella;* (also *Colobometra perspinosa*)	Free-living	Madang, Bismarck Sea, Papua New Guinea	**KM014206**	**KM014247**	**KM014288**	**KM014332**
Myzostoma testudo	SIO-BIC A3828	*Davidaster discoideus;* (also *Davidaster rubiginosus*)	Free-living	Carrie Bow Cay, Caribbean Sea, Belize	**KM014194**	**KM014237**	**KM014277**	**KM014320**
Myzostoma cf. *viride*	SIO-BIC A3808	*Comatella nigra* type B	Free-living	Lizard Island Reef, Coral Sea, Australia	**KM014187**	**KM014231**	**KM014270**	-
Myzostoma sp. A	-	*Phanogenia typica*	Free-living	Madang, Bismarck Sea, Papua New Guinea	**KM014205**	**KM014246**	**KM014287**	**KM014331**
Myzostoma sp. B	SAM E3395	*Phanogenia typica*	Free-living	Lizard Island Reef, Coral Sea, Australia	DQ238212	DQ238179	DQ238146	-
Myzostoma sp. C	SIO-BIC A3827	*Capillaster multiradiatus* s.l. B	Free-living	East Johor Strait, Singapore	**KM014212**	-	**KM014292**	**KM014336**
Myzostoma sp. D	SIO-BIC A3814	*Dichrometra/Lamprometra/Liparometra* sp. 2	Free-living	Stradbroke Island, Queensland, Australia	**KM014199**	**KM014241**	**KM014282**	-
Myzostoma sp. E	SIO-BIC A3720	Colobometridae sp.	Free-living	Madang, Bismarck Sea, Papua New Guinea	**KM014207**	**KM014248**	-	**KM014333**

Myzostoma sp. F	SIO-BIC A3672	*Oligometra serripinna;* (also *Petasometra clarae*)	Free-living	Madang, Bismarck Sea, Papua New Guinea	**KM014190**	**KM014234**	**KM014273**	**KM014316**
Myzostoma sp. G	SIO-BIC A3826	*Heterometra crenulata*	Free-living	East Johor Strait, Singapore	**KM014211**	**KM014249**	-	**KM014335**
Notopharyngoides aruensis	SIO-BIC A3817	*Stephanometra indica*	In digestive tube	Lizard Island Reef, Coral Sea, Australia	**KM014180**	**KM014225**	**KM014263**	-
Notopharyngoides platypus	SIO-BIC A3811	*Comaster audax;* (also *Comaster schlegelii*)	In cysts	Lizard Island Reef, Coral Sea, Australia	**KM014181**	**KM014226**	**KM014264**	**KM014305**
**Protomyzostomatidae** Stummer-Traunfels, 1923								
Protomyzostomum lingua	SIO-BIC A3238	*Gorgonocephalus chilensis*	Internal	Discovery Bank, Antarctica	**KM014171**	**KM014215**	**KM014253**	**KM014296**
Protomyzostomum roseus	SIO-BIC A3799	*Asteronyx longifissus*	Internal	Monterey, California, USA	**KM014172**	**KM014216**	**KM014254**	-
**Pulvinomyzostomatidae** Jägersten, 1940								
Pulvinomyzostomum inaki	SIO-BIC A1408/A1579	*Antedon* sp.	In mouth	Jaco Scarp, Costa Rica	**KM014173**	**KM014217**	**KM014255**	**KM014297**
Pulvinomyzostomum messingi	SIO-BIC A3800	*Gephyrocrinus messingi*	Free-living	Oregon	**KM014174**	**KM014218**	**KM014256**	**KM014298**
Pulvinomyzostomum pulvinar	?	*Leptometra phalangium*	In mouth	Banyuls-sur-Mer (Mediterranean Sea, France)	DQ238180	DQ238150	DQ238114	-

AM, Australian Museum, Sydney, Australia; **MNHN**, Museum national d'Histoire naturelle, Paris, France; **SAM**, South Australian Museum, Adelaide, Australia; **SIO-BIC**, Benthic Invertebrate Collection, Scripps Institution of Oceanography, La Jolla, CA.

Sequencing of fresh material suggests the following sequences currently available on Genbank and published in Lanterbecq et al. (2006) are erroneous, wholly or in part: (**18S**) –*Mesomyzostoma katoi* DQ238121 (has an erroneous insertion), *Protomyzostomum glanduliferum* DQ238148 (identical to partial fragments of *M. katoi*), *Protomyzostomum polynephris* DQ238149 (identical to partial fragments of *M. katoi*), *Asteromyzostomum* sp. DQ238147 (similar to *M. mortenseni*), *Myzostoma nigromaculatum* DQ238140 (similar to *Myzostoma coriaceum* – here considered sp. F, see below); **(COI)** *Myzostoma ambiguum* DQ23820 (similar to *M. mortenseni*).

Identifications are emended for the following Genbank entries: DQ238192, DQ238163, & DQ238128 – *Endomyzostoma clarki* (previously *Endomyzostoma tenuispinum*) all identical to *E. clarki* sequences; DQ238189, DQ238160, & DQ238125 – *Endomyzostoma clarki* (previously *Endomyzostoma* sp. 2) all identical to *E. clarki* sequences; DQ238191, DQ238162, & DQ238127 – *Eenymeenymyzostoma cirripedium* (previously *Endomyzostoma* sp. 3); DQ238212, DQ238179, & DQ238146 – *Myzostoma* sp. B (previously *Myzostoma coriaceum*); DQ238201, DQ238172, & DQ238136 – *Myzostoma polycyclus* (previously *Myzostoma toliarense*).

Table S2. Summary of maximum parsimony and maximum likelihood analyses.

Dataset	No. characters	No. parsimony informative sites	No. variable uninformative sites	No. of most parsimonious trees	Most parsimonious tree length	Best scoring maximum likelihood tree (-ln)
**Myzostomida**						
COI	621	322	26	4	3676	14987.78
COI -3rd position removed	414	120	21	13006	833	47532.15
16S	466	233	40	464	1622	7318.92
16S Gblocked	408	208	40	208	1502	6831.37
18S	1829	352	138	116	1712	11436.89
18S Gblocked	1678	312	102	108	1447	10055.26
H3	449	134	9	116	1114	5012.06
H3 Gblocked	335	134	9	113	1114	4851.35
**Hosts**						
Full concatenated dataset	5472	1477	487	4	7430	38387.23
Concatenated dataset – no 3rd positions and Gblocks	4117	879	394	18	3292	21198.34
COI	1062	423	73	35	2906	13523.09
COI -3rd position removed	708	124	44	76353	483	3417.55
16S	660	326	74	6	1594	7580.28
16S Gblocked	519	258	55	16	1190	5902.19
CytB	768	325	86	2	1729	8105.98
CytB -3rd position removed	512	112	64	24	497	2981.79

18S	2201	145	142	225	362	5014.41
18S Gblocked	1654	131	127	145	320	4053.67
28S	781	258	112	30	772	4597.97
28S Gblocked	724	254	104	36	757	4456.87
**Myzostomida Reduced**						
Full concatenated dataset	3343	1012	199	10	7751	36377.04
Concatenated dataset – no 3rd positions and Gblocks	2835	759	156	31	4727	25292.33
COI	621	318	27	6	3398	13963.00
COI -3rd position removed	414	118	20	9563	772	4025.87
16S	466	218	40	18	1497	6797.35
16S Gblocked	519	258	55	16	1228	6337.89
18S	1807	342	123	324	1519	10502.53
18S Gblocked	1678	304	96	12	1290	9308.28
H3	449	134	9	77	1110	4991.69
H3 Gblocked	335	134	9	77	1110	4829.42

Table S3. GenBank accession numbers for hosts and non-myzosome annelid outgroups used in molecular analyses. New molecular data in bold.

Host Taxa	Voucher	COI	16S	CytB	18S	28S
**Asteroidea**						
Labidiaster annulatus	GB	-	AY706154	-	AY935548	DQ354297
**Ophiuroidea**						
Gorgonocephalus chilensis	GB	AB758812	AB758495	-	AB758525	-
Asteronyx longifissus	SIO-BIC E6108	-	**KM014337**	-	-	**KM014340**
**Hyocrinida**						
Gephyrocrinus messingi	SIO-BIC E5662	**KM014350**	**KM014339**	-	-	**KM014342**
**Isocrinida**						
Endoxocrinus parrae	GB	GU327840	GU327875	GU327909	Z80951	GU327947
Metacrinus cf. *rotundus*	GB	KC626566	KC626658	-	KC626752	KC626846
Metacrinus levii	GB	GQ913322	GU327877	-	KC626751	GU327948
**Antedonidae**						
Antedon bifida	GB	KC626510	KC626604	-	KC626696	KC626791
Antedon sp.	SIO-BIC E4399	**KM014345**	**KM014338**	**KM014343**	-	**KM014341**
Florometra mawsoni	GB	KC626549	KC626641	-	KC626735	KC626829
Promachocrinus kerguelensis	GB	DQ823276	GU327886	DQ823333	GQ913342	JQ340282
**Comatulidae**						
Alloeocomatella pectinifera	GB	GU327852	GU327892	GU327921	-	GU327961
Alloeocomatella n. sp.	GB	KJ874980	KJ875023	KJ875162	-	KJ875199
Anneissia bennetti	GB	GQ913314	KJ875057	KJ875190	GQ913330	KJ875236
Capillaster multiradiatus s.l. A	GB	KJ874982	KJ875025	KJ875163	-	KJ875201
Capillaster multiradiatus s.l. B	GB	KJ874984	KJ875026	KJ875164	-	KJ875251
Cenolia trichoptera	GB	GU327854	GU327894	GU327922	-	GU327962
Clarkcomanthus albinotus	GB	KJ874987	KJ875035	KJ875171	GQ913328	KJ875212
Clarkcomanthus alternans	GB	KJ874993	KJ875035	KJ875171	-	KJ875212
Clarkcomanthus luteofuscum	GB	KJ874989	KJ875031	KJ875168		KJ875208
Clarkcomanthus mirabilis	GB	GQ913313	KJ875039	KJ875175	GQ913329	KJ875216
Clarkcomanthus mirus	GB	KJ875016	KJ875059	KJ875192	-	KJ875239
Comactinia titan	GB	KJ874992	KJ875034	-	-	KJ875211
Comanthus gisleni	GB	GU327856	KJ875037	KJ875173	GU327935	KJ875214
Comanthus parvicirrus	GB	KJ874997	KJ875040	KJ875176	-	KJ875217
Comanthus cf. *suavia*	GB	KJ875000	KJ875043	-	-	KJ875220
Comanthus sp. 4	GB	KJ874996	KJ875038	KJ875174	-	KJ875215

Comaster audax	GB	KJ875002	KJ875046	KJ875181	-	KJ875223
Comaster schlegelii	GB	KJ875004	KJ875048	KJ875182	GQ913333	KJ875225
Comatella nigra type A	GB	GU327858	GU327896	GU327924	-	GU327965
Comatella nigra type B	GB	KJ875006	KJ875050	-	-	KJ875227
Davidaster discoideus	GB	KJ875012	KJ875055	KJ875188	-	KJ875233
Davidaster rubiginosus	GB	KJ875013	KJ875056	KJ875189	-	KJ875234
Phanogenia gracilis sl. type A	GB	KJ875018	KJ875062	KJ875195	-	KJ875241
Phanogenia typica	GB	KJ875017	KJ875061	KJ875194	-	KJ875240
**Mariametroidea**						
Amphimetra tessellata	SAM K2028	**KM014354**	-	-	-	-
Basilometra boschmai	SIO-BIC E6072	**KM014346**	-	-	-	-
Cenometra bella	GB	GU327851	GU327890	GU327920	-	GU327959
Colobometra perspinosa	GB	GQ913321	GU327891	GU327919	GQ913338	GU327960
Colobometridae sp.	MNHN IE-2013-8040 SIO-BIC E6036*	**KM014348**	-	-	-	-
Dichrometra/Lamprometra/Liparometra sp. 2	SAM K2109	**KM014355**	**-**	**-**	-	-
Heterometra crenulata	GB	KC626554	KC626646	-	KC626740	KC626834
Himerometra robustipinna	GB	GQ913326	GU327898	GU327925	GQ913343	GU327970
Dichrometra/Lamprometra/Liparometra sp. 1	MNHN-IE-2013-8128 SIO-BIC E5912*	**KM014351**	-	-	-	-
Dichrometra/Lamprometra/Liparometra sp. 4	GB	GQ913319	GU327900	GU327927	GQ913335	GU327972
Oligometra serripinna	MNHN-IE-2013-8101 SIO-BIC E6034*	**KM014352**	-	-	-	-
Petasometra clarae	SIO-BIC 6071	**KM014347**	-	-	-	-
Pontiometra anderson	SIO-BIC E6070	**KM014344**	-	-	-	-
Stephanometra indica	GB	GQ913320	-	-	GQ913336-7	GU327973
Stephanometra tenuipinna	SIO-BIC E5842	**KM014353**	-	-	-	-
**Ptilometridae**						
Ptilometra macronema	GB	GU327866	GU327903	-	-	GU327976
**Tropiometridae**						
Tropiometra afra	GB	GU327867	GU327906	GU327928	-	GU327978
**Zenometridae**						
Psathyrometra fragilis	GB	GU327865	GU327905	-	-	-

**Host Outgroups**						
Asterias amurensis	GB	AB183559	AB183559	AB183559	D14358	-
Ophiopholis aculeata	GB	AF314589	DQ297105	AF314589	DQ060806	DQ029078
**Annelid Outgroups**						
Nereis pelagica	GB	JN852947	AY340470	-	AF474279	-
Glycera alba	GB	JN852946	DQ779615	-	AY176287	-
Bhawania heteroseta	GB	EU555053	EU555044	-	EU555035	-
Harmothoe imbricata	GB	AY839580	AY340463	-	AY340434	-
Chloeia flava	GB	JN852944	JN852917	-	EF076780	-
Phyllodoce maculata	GB	AY839586	-	-	AY176302	-
Eusyllis blomstrandi	GB	EF123749	EF123788	-	EF123887	-

MNHN, Museum national d'Histoire naturelle, Paris, France; **SAM**, South Australian Museum, Adelaide, Australia; **SIO-BIC**, Benthic Invertebrate Collection, Scripps Institution of Oceanography, La Jolla, CA.
